# Reversal of the seasonality of temperature-attributable mortality from respiratory diseases in Spain

**DOI:** 10.1038/s41467-020-16273-x

**Published:** 2020-05-20

**Authors:** Hicham Achebak, Daniel Devolder, Vijendra Ingole, Joan Ballester

**Affiliations:** 1 Centre for Demographic Studies (CED), Autonomous University of Barcelona (UAB), Barcelona, Spain; 2 Climate and Health Program (CLIMA), Barcelona Institute for Global Health (ISGlobal), Barcelona, Spain

**Keywords:** Climate-change impacts, Projection and prediction, Respiratory tract diseases, Epidemiology

## Abstract

A growing number of epidemiological studies have recently assessed temporal variations in vulnerability and/or mortality attributable to hot and cold temperatures. However, the eventual changes in the seasonal distribution of temperature-attributable mortality remain unexplored. Here, we analyse countrywide daily time-series of temperature and mortality counts from respiratory diseases by sex, age group and province of residence during the period 1980–2016 in Spain. We show the complete reversal of the seasonality of temperature-attributable mortality, with a significant shift of the maximum monthly incidence from winter to summer, and the minimum monthly incidence from early and late summer to winter. The reversal in the seasonal distribution of the attributable deaths is not driven by the observed warming in both winter and summer temperatures, but rather by the very large decrease in the risk of death due to cold temperatures and the relatively much smaller reduction due to hot temperatures. We conclude that the projected decrease in the number of moderate and extreme cold days due to climate warming will not contribute to a further reduction of cold-attributable respiratory deaths.

## Abstract (web summary)

Potential changes in the seasonality of temperature-attributable mortality due to climate warming have been poorly investigated. Here, the authors show that the projected decrease in the number of moderate and extreme cold days will not contribute to a further reduction of cold-attributable deaths.

## Introduction

Human-driven climate change has become a major concern for public health worldwide^1^. Its direct impacts on health expand across a range of sectors^2^, including changes in mortality and morbidity rates associated with the general rise in temperatures and the related increase in the frequency, intensity and duration of extreme heatwaves^3^.

Projections of the impact of rising temperatures on mortality consistently indicate a progressive increase in heat-attributable mortality and a decrease of cold-attributable mortality during the next decades, resulting in a substantial positive or negative net effect in temperature-attributable mortality depending on the location and magnitude of the warming^4^. However, this scenario is subject to a high level of uncertainty, given that it will also depend on the future capacity of the societies to reduce their vulnerability to both warm and cold temperatures^5^. Trends in the health impact (i.e. heat-attributable and cold-attributable deaths) essentially arise from the combination of variations in both exposure and vulnerability. A decrease in vulnerability, often expressed as relative risk (RR)^6^, can be largely linked to adaptation processes, either occurring by means of a physiological acclimatisation response of the population to changing temperature (intrinsic or causal adaptation)^7^, or independently from the warming through a range of non-climate driven factors (extrinsic adaptation), such as socioeconomic development or improved healthcare services^8^.

A growing number of epidemiological studies have recently assessed temporal variations in the vulnerability and/or mortality attributable to hot and cold temperatures, reporting evidence of a reduction in population vulnerability to both heat and cold in some, albeit not all, of the analysed countries^7,9–12^. However, even though it is well established that the impact of temperature on mortality varies by season in extratropical countries, being higher in winter than during other parts of the year^13^, mainly for cardiovascular and respiratory diseases, the possible change in the seasonal distribution of temperature-attributable mortality remains unexplored.

In the present work, we examine trends in the seasonality of temperature-attributable mortality from respiratory diseases by sex and age group between 1980 and 2016 in Spain. Ambient temperatures are mainly correlated with cardiovascular and respiratory diseases, and the mechanisms behind these associations, as well as their recent evolution, are substantially different^6^. We here specifically analyse mortality from respiratory causes, and discuss differences with regard to recent findings on cardiovascular diseases^12^. Results show a very strong reduction in the temperature-attributable fraction (AF) during the coldest months of the year and only a small decrease during the hottest ones, resulting in the redefinition of the seasonality of mortality, with a shift of the maximum monthly AF from winter to summer, and the minimum monthly AF from early and late summer to winter. We conclude that the projected decrease in the number of moderate and extreme cold days will not contribute to a further reduction of cold-attributable respiratory deaths in Spain, which opens a new avenue towards a more realistic estimation of future mortality under climate change scenarios.

## Results

### Evolution of mortality and temperatures

We analysed data from 48 provinces in mainland Spain and the Balearic Islands (Supplementary Fig. 1), which included 1,306,283 deaths from respiratory diseases (10.9% of total mortality due to natural causes), covering a period of 37 years from 1980 to 2016. The number of deaths from respiratory causes showed an important increase over the study period for both men (+66.5%) and women (+77.3%), except for the age group 60–74 years (Supplementary Fig. 2). The proportion of mortality due to respiratory causes (i.e. ratio between respiratory and total deaths) has risen from 9.9% in 1980 to 11.9% in 2016, representing a relative increase of 20.2%. In parallel, the distribution of temperatures has shifted towards higher values, generally with more moderate and extreme warm days and less moderate and extreme cold days in 2002–2016 compared with 1980–1994 (Supplementary Fig. 3).

### Risk of death due to temperatures

The RR values associated with the temperature–mortality relationships by sex and age group for the whole study period indicate that both low and high temperatures are associated with increased risk of mortality, especially in the case of the extreme temperatures (Supplementary Fig. 4). The heat slope was in all cases much steeper than the cold one, and varied greatly by sex and age group. Thus, the heat slope of women and the older age groups was higher than the ones for men and the younger age groups, respectively. By contrast, the cold slope was slightly more pronounced for men than for women, and for younger age groups than for older ones for cold temperatures. The point of MMT decreased with age, and it was higher for men than for women.

**Fig. 1:**
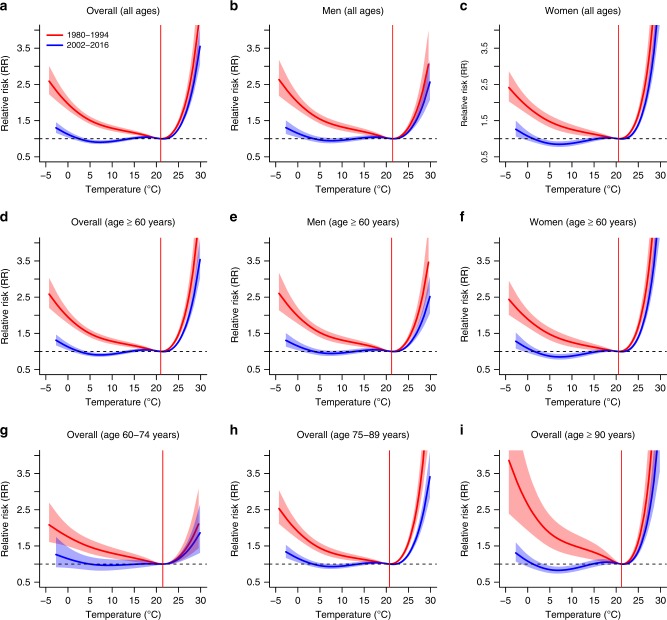
Relative risk (unitless) of death from respiratory diseases in Spain. The minimum mortality temperature in 1980–1994 is used as the centring temperature for the two subperiods (i.e. *invariant centring temperature assumption*, see red vertical lines).

Temporal changes in the pooled exposure-response relationships between temperature and mortality are displayed in Fig. 1 (see province-specific estimates in Supplementary Figs. 5–10). The RR curves, which are all centred at the MMT value of the 1980–1994 subperiod (i.e. *invariant centring temperature assumption*, see the red vertical line in the panels), suggest a very strong reduction in the effects of cold on mortality for all the sex and age groups, while the reductions in the mortality risk associated with heat were generally smaller. Specifically, the RR corresponding to the 1st temperature percentile of the whole study period fell from 1.805 (95% empirical CI: 1.670–1.952) in 1980–1994 to 1.047 (0.973–1.126) in 2002–2016, and the RR corresponding to the 99th percentile from 1.885 (1.696–2.095) to 1.610 (1.520–1.705). Overall, very small differences in the RR due to cold temperatures are found among sex and age groups in the last subperiod, showing an almost complete process of adaptation to cold, while differences are still significant in summer.

### Trends in minimum mortality temperature

**Fig. 2:**
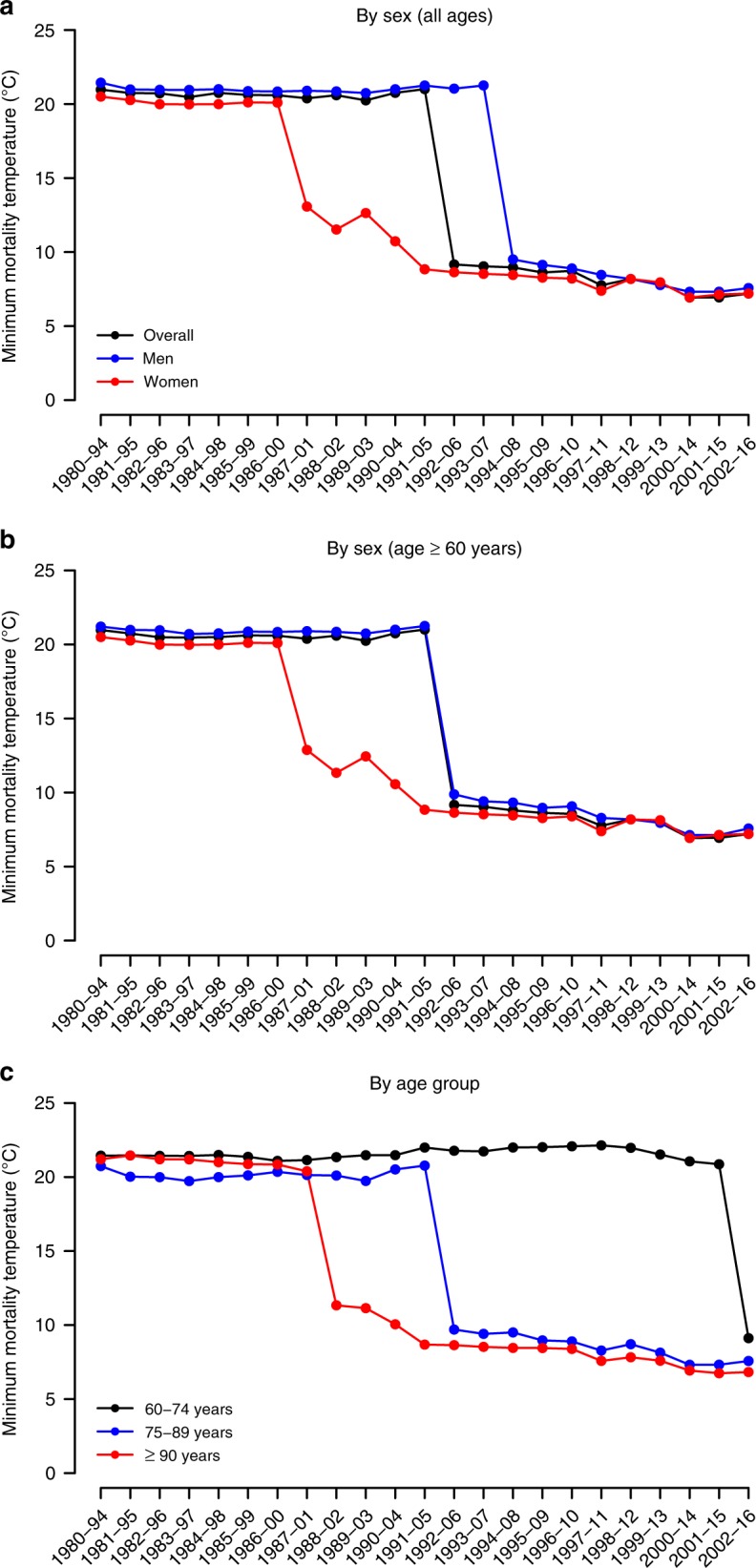
Minimum mortality temperature (°C) corresponding to respiratory diseases.

The curves in Fig. 2 depict the estimates of minimum MMT extracted from exposure–response curves computed from the 23 subperiods of 15 consecutive years. MMT largely cooled both for men and women, as well as for all the age groups. The overall MMT fell from 21.0 °C in 1980–1994 to 7.2 °C in 2002–2016, which corresponds to a displacement from percentiles 83 (i.e. in summer) to 18 (in winter) of the daily temperature distribution of the whole study period. Furthermore, the shift in the MMT occurred earlier in women than in men, and in the older age groups than in the younger ones. However, given that the RR curves changed from an asymmetric V-shape to a hockey stick, with flat values for all non-summer temperatures (Fig. 1), the confidence interval of the MMT largely expanded towards the coldest values, and therefore, the temporal changes in the MMT are not statistically significant in most of the cases (Supplementary Figs. 11–22).

### Reversal of the seasonality of temperature-attributable mortality

**Fig. 3:**
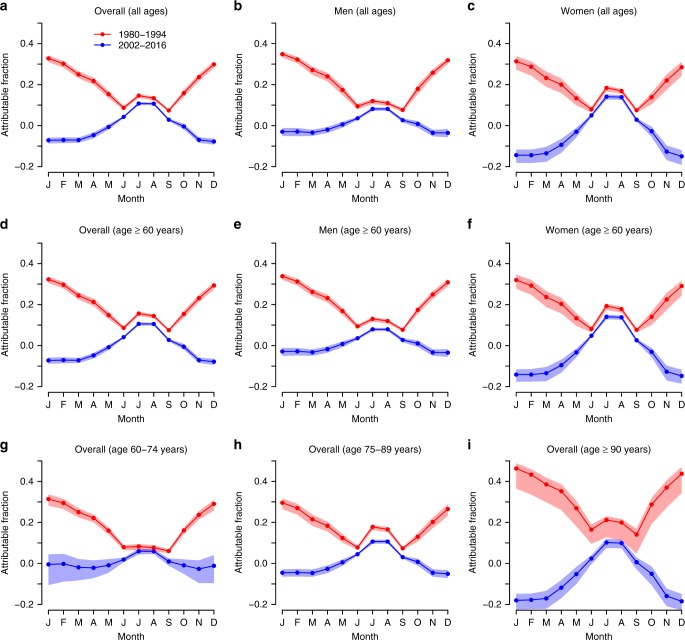
Monthly attributable fraction (unitless) of respiratory disease mortality in Spain. The minimum mortality temperature in 1980–1994 is used as the centring temperature for the two subperiods (i.e. *invariant centring temperature assumption*). The attributable fractions (AF) are estimated with the corresponding 95% empirical CI.

Figure 3 shows the AF by month of the year for the first and last 15-year subperiods of the series (see Supplementary Fig. 23 for other subperiods, and Supplementary Figs. 24–29 for province-specific estimates). Strikingly, the results reveal a complete change in the seasonality of the AF between 1980–1994 and 2002–2016 for both sexes and all the age groups, with a displacement of the maximum monthly AF from winter to summer, and the minimum monthly AF from early and late summer to winter. This statistically significant reversal of the seasonality has been essentially driven so far by the very large decrease in the RR due to cold temperatures and the modest decrease due to hot temperatures, and not by the observed warming in both winter and summer (Supplementary Fig. 30).

**Fig. 4:**
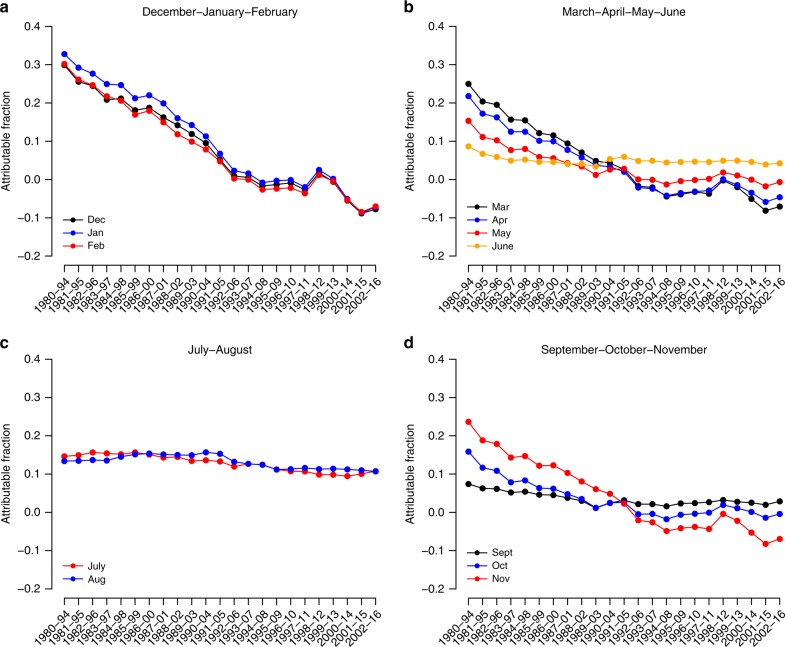
Evolution of the monthly attributable fraction (unitless) of respiratory disease mortality in Spain. The minimum mortality temperature in 1980–1994 is used as the centring temperature for all the subperiods (i.e. invariant centring temperature assumption).

**Fig. 5:**
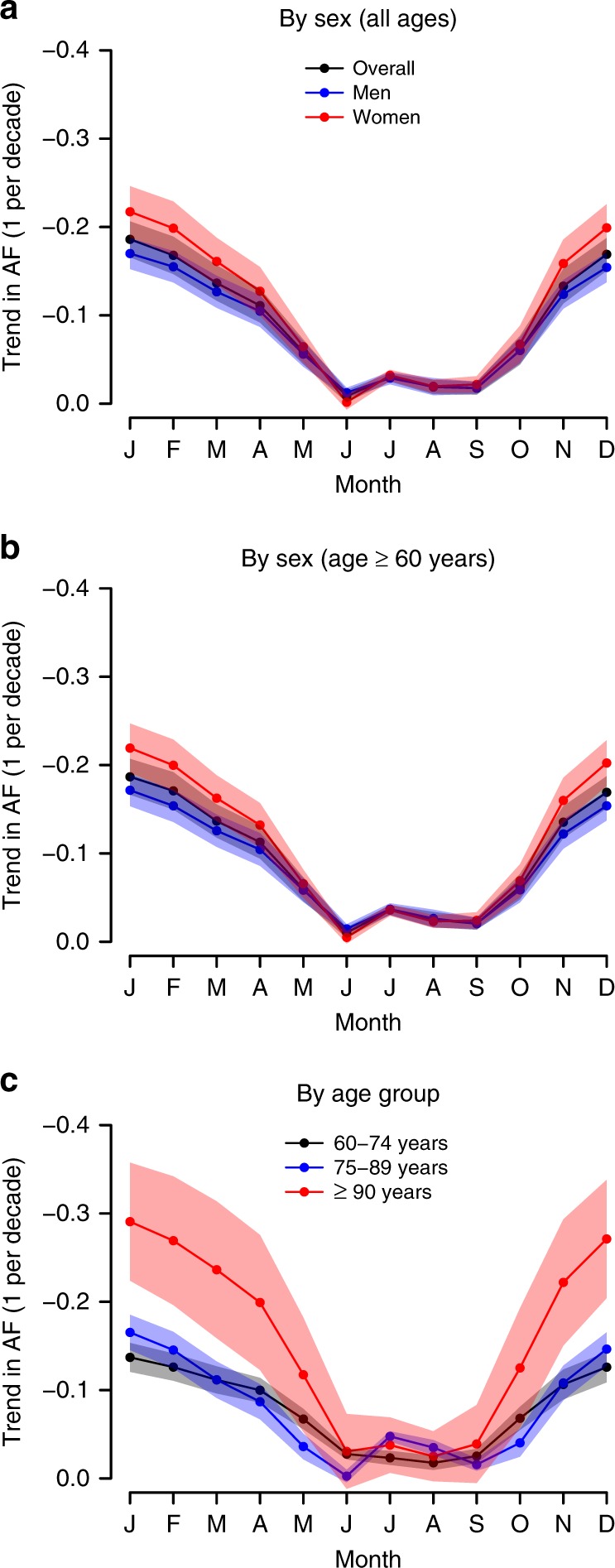
Trends in monthly attributable fraction (1 per decade) of respiratory disease mortality in Spain. The minimum mortality temperature in 1980–1994 is used as the centring temperature for all the subperiods (i.e. invariant centring temperature assumption). Data are presented as mean values ± 95% empirical CI.

The trends in overall monthly AF are summarised in Fig. 4, and indicate a steep decrease in the AF during the cold months of the year, especially in winter (December–March), and only a slight reduction during the summer months (June–September). For instance, the value of the trend in AF for the coldest month of the year (i.e. January) was −0.19 per decade (95% empirical CI: −0.17 to –0.21), whereas for the hottest one (i.e. August) was −0.02 per decade (−0.01 to −0.03) (Fig. 5). It is also noteworthy that, in line with the changes in the MMT, the monthly AF generally declined at a faster pace for women than for men, and for the older age groups than for the younger ones (Fig. 5).

### Invariant and varying centring temperature

All the results shown here have been reported under the *invariant centring temperature assumption*, that is, the RR at the MMT of the first subperiod does not change over time and is equal to 1 in all the subperiods. Please note that a similar assumption is implicitly used with time-varying distributed lag non-linear model^7,10,11^. Given the very strong decrease of the slope of the RR for the cold temperatures, this implicitly implies that the RR is in some cases smaller than 1 for some cold temperatures in the most recent subperiods (Fig. 1), and therefore, the AF can become negative (Fig. 3). This negative value should only be understood as a way to evaluate the trend (or more precisely, the relative inter-subperiod change) in the AF by taking the first subperiod as the reference baseline (Fig. 4), and not to quantify the actual absolute burden in a given subperiod. In the Supplementary Information (Supplementary Figs. 31–39), the same analyses are provided under the *varying centring temperature assumption*, that is, the centring temperature is allowed to change over time, and therefore, the RR is never smaller than 1 and the AF is never negative. This implicitly implies that the RR at the MMT of the first subperiod and the AF in summer have both increased. This scenario is here considered to be unrealistic because it results to be an artefact of the modelling procedure, which implicitly assumes an increase of the RR for some temperatures that is contrary to the available literature^6^.

All sensitivity analyses suggested that the reported results were not dependent on modelling assumptions (Supplementary Fig. 40).

## Discussion

This study reports a complete reversal of the seasonality of temperature-attributable mortality from respiratory diseases over the last four decades in Spain. Results of this investigation showed a very strong reduction in the AF during the coldest months of the year and only a small decrease during the hottest ones, resulting in the redefinition of the seasonality of mortality, with a shift of the maximum monthly AF from winter to summer, and the minimum monthly AF from early and late summer to winter. These findings have major implications for climate change health adaptation policies. We conclude that the projected decrease in the number of moderate and extreme cold days will not contribute to a further reduction of cold-attributable respiratory deaths in the country.

In this study, the decline in the vulnerability (expressed as RR) to cold temperatures was so large that the MMT associated with respiratory diseases moved from very warm to very cold temperatures. This cooling in the MMT contrasts with the warming trend found in previous studies for other causes of death^14,15^. For instance, in Spain, the MMT for cardiovascular diseases rose from 19.5 °C in 1980–1994 to 20.2 °C in 2002–2016, a warming that was similar in magnitude, and occurred in parallel with, the recorded average warming of 0.77 °C between these two periods^12^. Moreover, the MMT, which typically ranges between the 60th and the 90th temperature percentile, is commonly used as the reference point to separate between heat- and cold-attributable mortality^16^. Given the abrupt cooling of the MMT here observed, a definition of heat and cold from a health perspective, i.e. based on this optimum point, does not seem a good choice for the characterisation of trends in attributable mortality, and therefore we favoured the description of trends by monthly attributable values.

We found a change in the seasonal distribution of temperature-attributable mortality from respiratory diseases. This was essentially explained by the large differences in the reduction of the RR for the cold and hot temperatures. The large decline in the RR associated with cold temperatures contributed to a steep decrease in the AF in the coldest moths of the year, while the smaller reduction in the RR associated with warm temperatures only contributed to a limited reduction in the AF in the hottest months. In earlier investigations conducted in the United States^9^ and Japan^17^, the rate of decline in heat-related respiratory mortality during summer was larger than in our study, whereas a comparison for cold temperatures is not possible due to the lack of enough evidence in the literature.

The general reduction in the vulnerability and/or impact due to heat has been attributed in many studies to socioeconomic development and structural transformations, such as improvements in housing conditions and healthcare systems (e.g., improved treatment of heat-related morbidity)^11,17^, the reduction in risk factors (e.g. smoking and healthier diet)^9^, or even to planned adaptation policies led by governments and public health agencies^18,19^. In Spain, the large socioeconomic advances experienced during the study period might have widely contributed to the declining vulnerability to heat and, especially, cold temperatures. For example, the gross domestic product increased from €8789 per capita in 1991 to €22,813 in 2009, and the expenditure in healthcare system from €605 per capita to €2182 (ref. ^20^). In addition, the percentage of households with air conditioning increased from 4.16% in 1991 (ref. ^21^) to 35.5% in 2008 (ref. ^22^), and with central heating from 25.83% in 1991 to 56.86% in 2011 (ref. ^21^).

This study also revealed differences in the effects of hot and cold temperatures on respiratory deaths by sex and age. Mortality risks due to heat were higher for women than for men and increased with age, while, on the contrary, the effects of cold were lower for women than for men and decreased with age. Findings of previous studies on sex and age differences have been mixed. Some articles have shown that men were more sensitive to heat than women^23,24^, while other studies reported the opposite result^6^. This heterogeneity may arise from socioeconomic, cultural and health-related factors. With respect to the effect modification by age, the elderly has been described as the most vulnerable population group for heat^17,23,25^, while differences by age for cold temperatures are not homogeneous^26^. For instance, an acute effect of cold on respiratory mortality was observed for subjects aged less than 65 years in the United States^27^. One possible explanation for that pattern is that the elderly tend to stay indoors more often than younger people, with more limited social contact, and thus avoid direct exposure to cold temperatures and infectious diseases. The underlying physiological mechanisms by which heat and cold trigger respiratory mortality are not well understood, but they seem to be largely mediated by a thermoregulatory pathway^28^.

Finally, this study has three main limitations that need to be mentioned. First, we were not able to control for ambient air pollution in the models because of data unavailability, and therefore, we do not know the extent to which this would have affected mortality trends here reported. However, the available literature on the confounding effect of air pollution on temperature–mortality associations shows modest^27,29^ or no modifying effect^30,31^. Second, the multivariate random-effect meta-analysis used in our study to drive and pool estimates from the multi-location DLNM does not consider the non-random spatial dependence of mortality, which could produce biased estimates. However, demographic and socio-economic characteristics related to mortality in Spain do not differ greatly between regions. Third, the present work did not describe the drivers of the observed change in the seasonality of temperature-attributable respiratory mortality in Spain, which will be addressed in a future study including socioeconomic and demographic data.

## Methods

### Data collection

Countrywide time-series of daily mortality counts from respiratory diseases as primary cause of death disaggregated by sex, 15-year age groups (0–14, 15–29, …, 75–89, ≥90 years) and province of residence between the years 1980 and 2016 were provided by the Spanish National Statistics Institute (INE). Note that the coding of death certificates changed during the study period from ICD-9 (460–519) in 1980–1998 to ICD-10 (J00–J99) in 1999–2016, although both classifications contained the same disaggregation of causes of death. In addition, daily high-resolution gridded (0.25° × 0.25°) observations of daily mean 2-m temperature were derived from E-OBS v16 of the European Climate Assessment and Dataset (ECA&D), and transformed into regional estimates using the average temperature for each province^32^. Both mortality and temperature datasets had no missing values.

### First-stage time-series model

The statistical analysis was performed in two stages. In the first stage, standard quasi-Poisson regression models allowing for overdispersion were individually applied to the whole study period (1980–2016) and data subsets of 15-year moving periods (1980–1994, 1981–1995, …, 2002–2016) in each of the 48 Spanish provinces to derive estimates of province-specific temperature–mortality associations, reported as RR by sex and age group. The models included a natural cubic B-spline of time, with 8 degrees of freedom (df) per year to adjust for the seasonality and the long-term trend, as well as a categorical variable to control for the day of the week. The temperature–mortality dependency was captured by using a distributed lag non-linear model (DLNM), which is based on the definition of a cross-basis function combining exposure–response and lag–response associations^33^. On the one hand, the exposure–response curve was modelled through a natural cubic B-spline with three internal knots placed at the 10th, 75th and 90th percentile of the daily temperature distribution. On the other hand, the lag-response curve was modelled through a natural cubic B-spline, with an intercept and three internal knots placed at equally spaced values in the log scale, and a lag period extending up to 21 days to account for the long-delayed effects of cold and short-term harvesting (i.e. deaths brought forward by only a few days due to temperature)^16^. In this way, the overall effect of a given day temperature on the RR of death was defined as the sum of the effects on that day and the 21 subsequent days. The quasi-Poisson regression model was given as follows:

Log*E* (*Y*) = intercept + *cb* + dow + *S*(time, d*f* = 8 × year),

where *Y* denotes the series of daily mortality counts; *cb* the cross-basis matrix produced by DLNM; dow the day of the week; and *S* the natural cubic B-spline of time. The modelling choices were thoroughly tested in sensitivity analyses by varying the number of knots in the exposure-response function, the number of lag days, and the number of degrees of freedom used to control for the seasonality and the long-term trend (Supplementary Fig. 40). We did not assess the associations between temperature and respiratory mortality specifically for individuals younger than 60 years because of the small number of daily deaths counts recorded for those age ranges in most provinces, which did not guarantee model convergence and optimal fitting. We did not include relative humidity into the analyses because its potential confounding effect was very small in past studies^34–36^. Furthermore, the use of combined indices of temperature and humidity, such as apparent temperature, did not predict mortality better than the single measure of temperature^37,38^, and the assessment of the effect of temperature and humidity separately showed that humidity plays a small and inconsistent role in affecting mortality^39^.

### Second-stage meta-analysis

In the second stage, a multivariate random-effects meta-analysis was used to estimate the mean RR values associated with the temperature–mortality curves across provinces^40^, and to derive the best linear unbiased prediction of the temperature–mortality associations in each location. We then extracted the minimum mortality temperature (MMT) from the country-level and province-specific exposure-response curves as the temperature with minimum RR.

### Centring temperature assumptions

Temporal changes in the exposure-response curves between 15-year subperiods were presented under two different centring temperature assumptions. Note that the centring temperature refers to the reference temperature value in which the RR is imposed to be equal to 1, and it does not need to coincide with the MMT. In the first case, here referred to as “*invariant centring temperature assumption*”, the value of the MMT obtained in the first 15-year subperiod (1980–1994) was chosen as the centring point of the exposure–response relationship in all the 15-year subperiods. This scenario therefore assumes that the RR at the MMT of the first subperiod does not change over time, and it therefore allows for RR values smaller than one. In the second case, shown in Supplementary Information (Fig. 31), the value of the MMT obtained in a given 15-year subperiod was automatically chosen as the centring temperature of the subperiod. This alternative scenario, here referred to as “*varying centring temperature assumption*”, assumes that the centring temperature changes over time, and therefore, the RR is never smaller than one. Note that the choice of the centring temperature only displaces the curve along the vertical RR axis, and therefore, it does not modify the shape of the model fit. However, the choice does modify the magnitude of the estimates in each subperiod, and therefore, the one of the long-term trends.

### Attributable risk

The mortality burden attributable to ambient temperatures, reported as the AF of deaths, was estimated by using the methodology developed by Gasparrini and Leone^41^. First, the overall RR corresponding to each day of the series was used to calculate the AF of deaths on that day and the next 21 days. Then, the daily attributable number (AN) of deaths was computed by multiplying the daily AF by the daily number of deaths. The number of AN in each month was separately aggregated from the daily series, and its ratio with the corresponding total number of deaths provided the monthly AF. We calculated 95% empirical CIs (eCIs) of attributable mortality using Monte Carlo simulations.

### Statistical software

All statistical analyses were done with R software (version 3.6.1) using functions from the packages *dlnm* (for the first-stage regression) and *mvmeta* (for the second-stage meta-analysis).

### Reporting summary

Further information on research design is available in the Nature Research Reporting Summary linked to this article.

## Supplementary information

Supplementary Information

Reporting Summary

## Data Availability

The data that support the findings of this study are available from the corresponding author upon reasonable request. The climate data can be obtained from the European Climate Assessment and Dataset (ECA&D, www.ecad.eu). The mortality data can be obtained from the Spanish National Statistics Institute (INE) under request. Unfortunately, we cannot publish the mortality data because several restrictions imposed by the INE apply. Specifically, the INE stipulates in the contract for the supply of the data the following clause: “No distribuir a terceros” (“Do not distribute the data to third parties” in English).

## Code availability

The code is available on request.
