# Supplementary material for: Reversal of the seasonality of temperature-attributable mortality from respiratory diseases in Spain
Source: Nat Commun. 2020 May 20;11:2457. doi: 10.1038/s41467-020-16273-x (PMC7239891; doi:10.1038/s41467-020-16273-x)
Supplement: Supplementary file 1 — Supplementary Information [file 41467_2020_16273_MOESM1_ESM.pdf]

SUPPLEMENTARY INFORMATION

Reversal of the seasonality of temperature-attributable mortality from respiratory diseases in Spain

Hicham Achebak, Daniel Devolder, Vijendra Ingole, Joan Ballester

Centre for Demographic Studies (CED), Autonomous University of Barcelona (UAB), Barcelona, Spain (HA, DD)
Climate and Health Program (CLIMA), Barcelona Institute for Global Health (ISGlobal), Barcelona, Spain (HA, VI, JB)

TABLE OF FIGURES

Supp. Figure 1: Provinces of Spain and the corresponding average daily mean temperature (°C), 1980-2016

Supp. Figure 2: Evolution of the number of deaths from respiratory diseases in Spain by age and sex, 1980-2016

Supp. Figure 3: Distribution of daily mean temperatures in Spain for June-September (A) October-May (B)

Supp. Figure 4: Relative risk of death from respiratory diseases in Spain for 1980-2015

Supp. Figure 5: Relative risk of death from respiratory diseases in the Spanish provinces, with related daily temperature distributions

Supp. Figure 6: Relative risk of death from respiratory diseases in the Spanish provinces, with related daily temperature distributions

Supp. Figure 7: Relative risk of death from respiratory diseases in the Spanish provinces, with related daily temperature distributions

Supp. Figure 8: Relative risk of death from respiratory diseases in the Spanish provinces, with related daily temperature distributions

Supp. Figure 9: Relative risk of death from respiratory diseases in the Spanish provinces, with related daily temperature distributions

Supp. Figure 10: Relative risk of death from respiratory diseases in the Spanish provinces, with related daily temperature distributions

Supp. Figure 11: Evolution of the overall minimum mortality temperature (°C) by province

Supp. Figure 12: Evolution of the overall minimum mortality temperature (°C) by province

Supp. Figure 13: Evolution of the overall minimum mortality temperature (°C) by province

Supp. Figure 14: Evolution of the overall minimum mortality temperature (°C) by province

Supp. Figure 15: Evolution of the overall minimum mortality temperature (°C) by province

Supp. Figure 16: Evolution of the overall minimum mortality temperature (°C) by province

Supp. Figure 17: Evolution of the minimum mortality temperature (°C) by sex and province

Supp. Figure 18: Evolution of the minimum mortality temperature (°C) by sex and province

Supp. Figure 19: Evolution of the minimum mortality temperature (°C) by sex and province

Supp. Figure 20: Evolution of the minimum mortality temperature (°C) by sex and province

Supp. Figure 21: Evolution of the minimum mortality temperature (°C) by sex and province

Supp. Figure 22: Evolution of the minimum mortality temperature (°C) by sex and province

Supp. Figure 23: Monthly Attributable Fraction of respiratory disease mortality in Spain by sex and age for 15-year subperiods

Supp. Figure 24: Monthly Attributable Fraction of respiratory disease mortality in the Spanish provinces

Supp. Figure 25: Monthly Attributable Fraction of respiratory disease mortality in the Spanish provinces

Supp. Figure 26: Monthly Attributable Fraction of respiratory disease mortality in the Spanish provinces

Supp. Figure 27: Monthly Attributable Fraction of respiratory disease mortality in the Spanish provinces

Supp. Figure 28: Monthly Attributable Fraction of respiratory disease mortality in the Spanish provinces

Supp. Figure 29: Monthly Attributable Fraction of respiratory disease mortality in the Spanish provinces

Supp. Figure 30: Monthly Attributable Fraction of respiratory disease mortality in Spain

Supp. Figure 31: Relative risk of death from respiratory diseases in Spain for the periods 1980-1994 and 2002-2016

Supp. Figure 32: Monthly Attributable Fraction of respiratory disease mortality in Spain by sex and age

Supp. Figure 33: Monthly Attributable Fraction of respiratory disease mortality in the Spanish provinces

Supp. Figure 34: Monthly Attributable Fraction of respiratory disease mortality in the Spanish provinces

Supp. Figure 35: Monthly Attributable Fraction of respiratory disease mortality in the Spanish provinces

Supp. Figure 36: Monthly Attributable Fraction of respiratory disease mortality in the Spanish provinces

Supp. Figure 37: Monthly Attributable Fraction of respiratory disease mortality in the Spanish provinces

Supp. Figure 38: Monthly Attributable Fraction of respiratory disease mortality in the Spanish provinces

Supp. Figure 39: Evolution of the monthly Attributable Fraction of respiratory disease mortality in Spain

Supp. Figure 40: Sensitivity analysis on the monthly Attributable Fraction of respiratory disease mortality in Spain

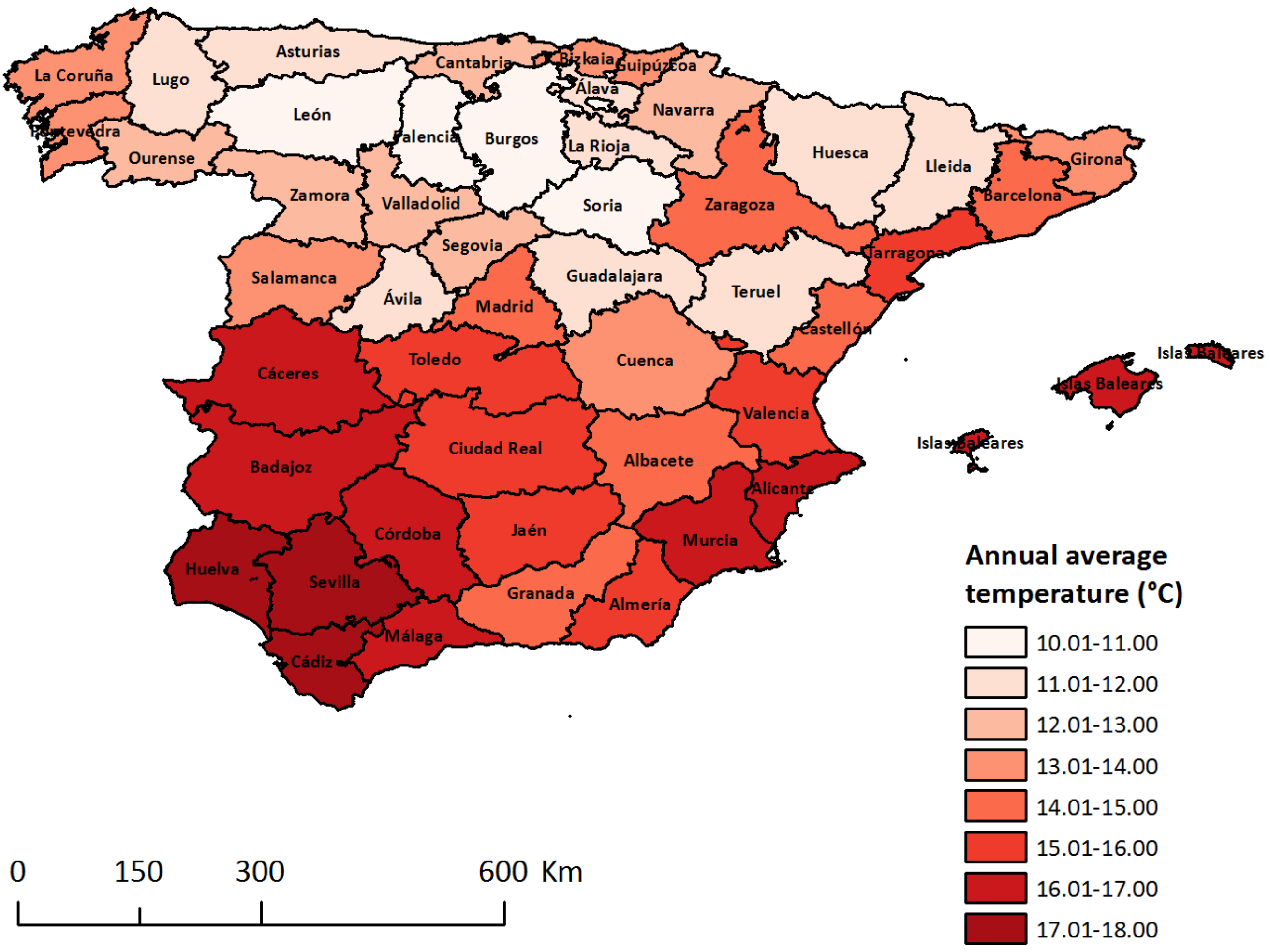

Supplementary Figure 1: Provinces of Spain and the corresponding average daily mean temperature (°C), 1980-2016

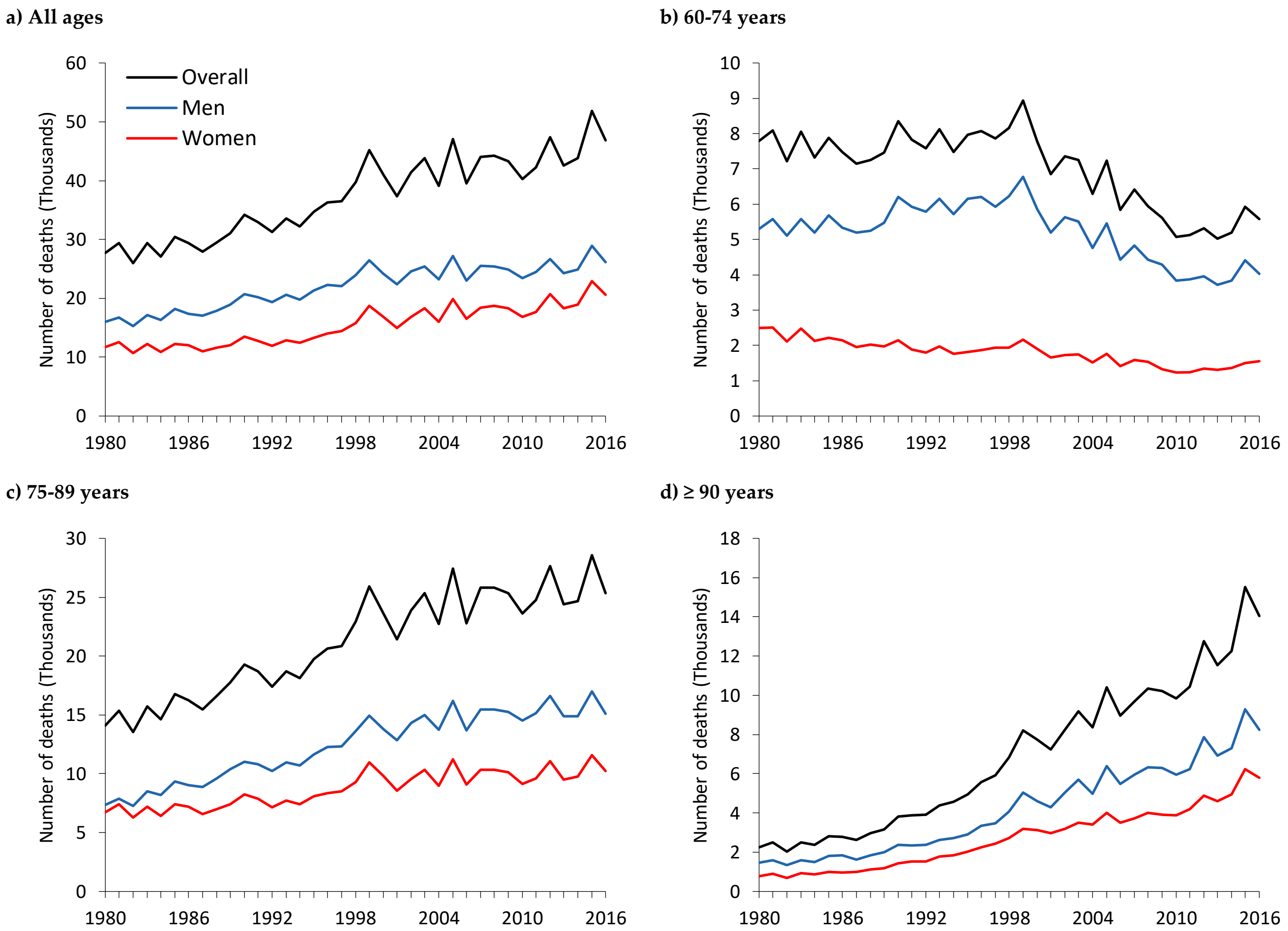

Supplementary Figure 2: **Evolution of the number of deaths from respiratory diseases in Spain by age and sex, 1980-2016.**

a) June-September

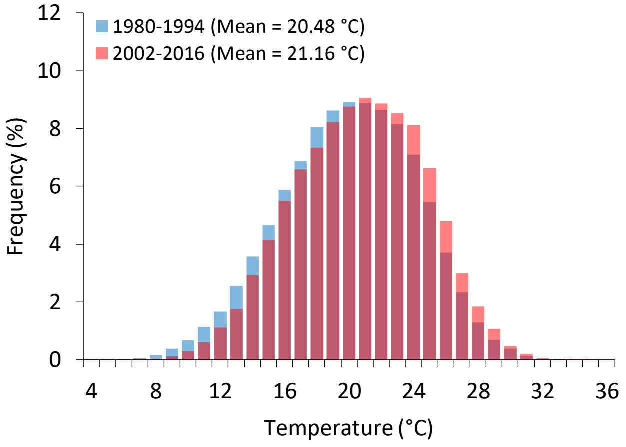

b) October-May

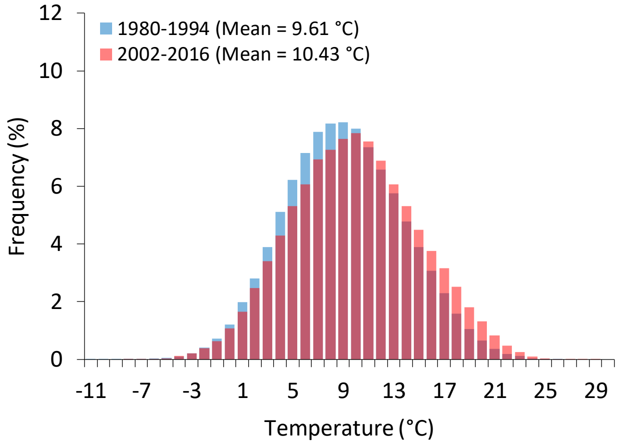

Supplementary Figure 3: **Distribution of daily mean temperatures in Spain for June-September (A) October-May (B).**

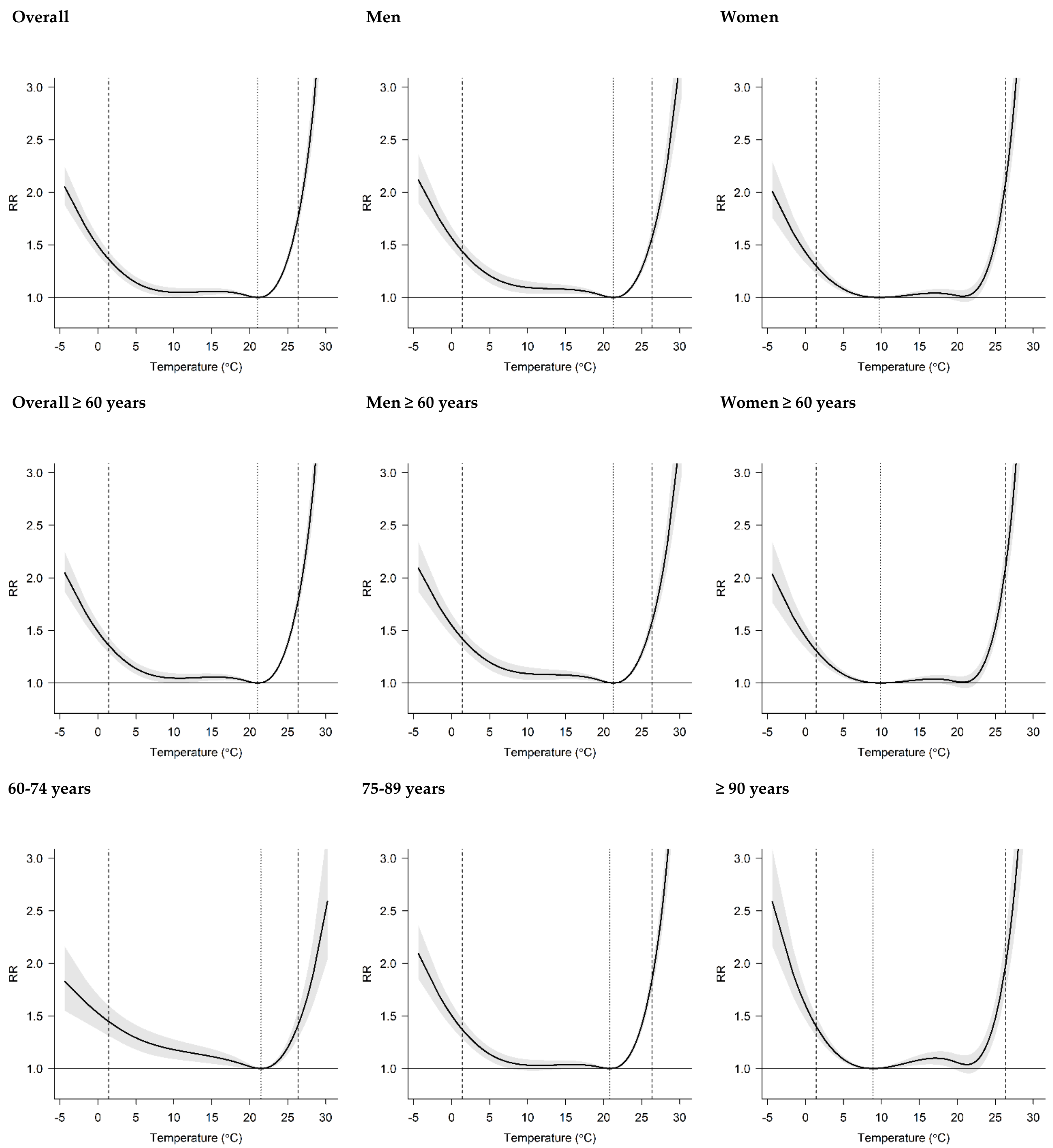

Supplementary Figure 4: **Relative risk of death from respiratory diseases in Spain for 1980-2015.** The relative risk (RR) curves are estimated with the corresponding 95% empirical CI. Vertical dotted lines are minimum mortality temperatures (MMTs), and dashed lines the 1st and 99th percentiles of the daily temperature distribution.

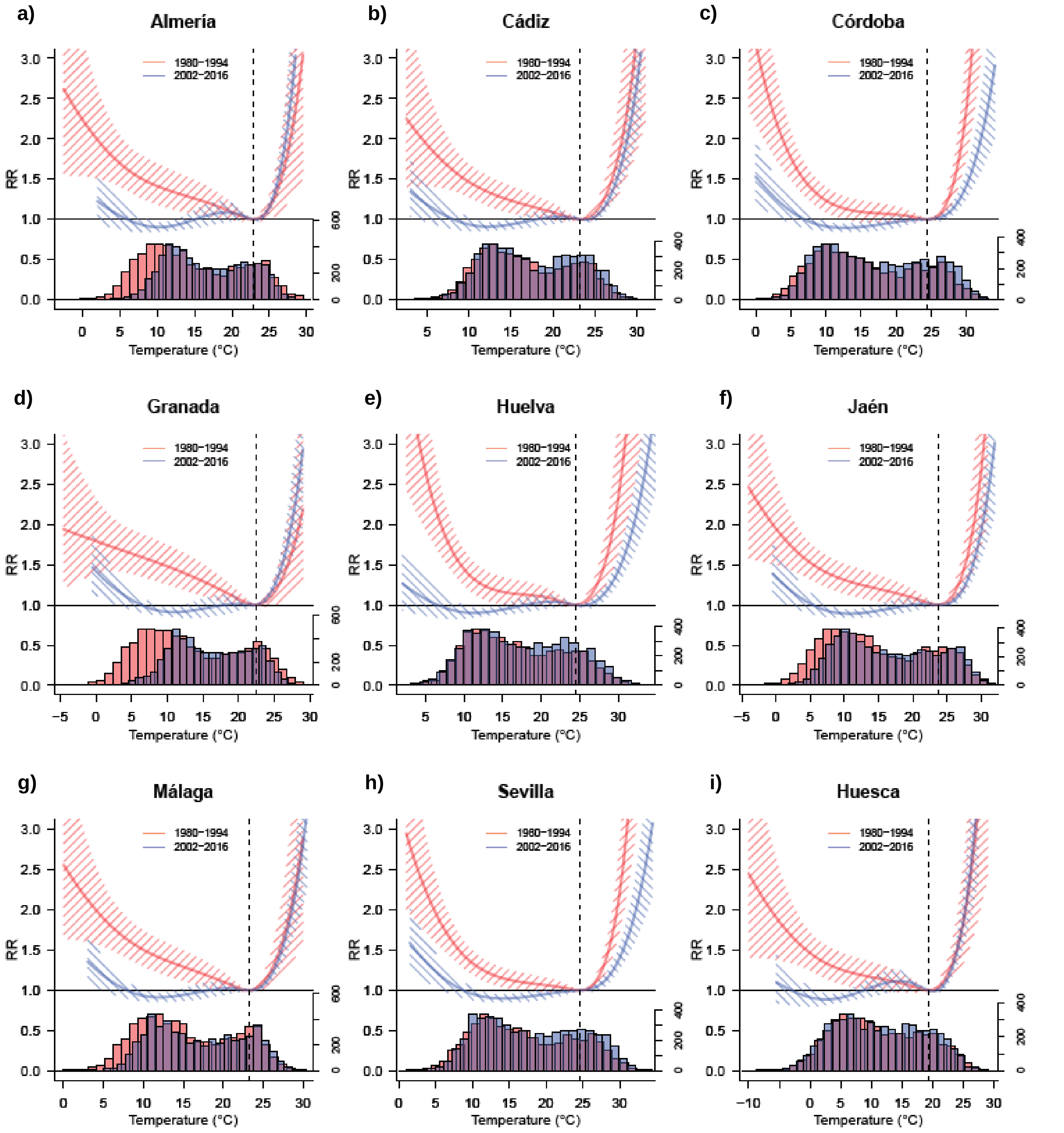

Supplementary Figure 5: **Relative risk of death from respiratory diseases in the Spanish provinces, with related daily temperature distributions.** The relative risk (RR) curves are estimated with the corresponding 95% empirical CI. The vertical dashed lines represent the Minimum Mortality Temperature (MMT) of the subperiod 1980-1994 is used as the centring temperature for the two subperiods ("*invariant centring temperature assumption*", see Methods).

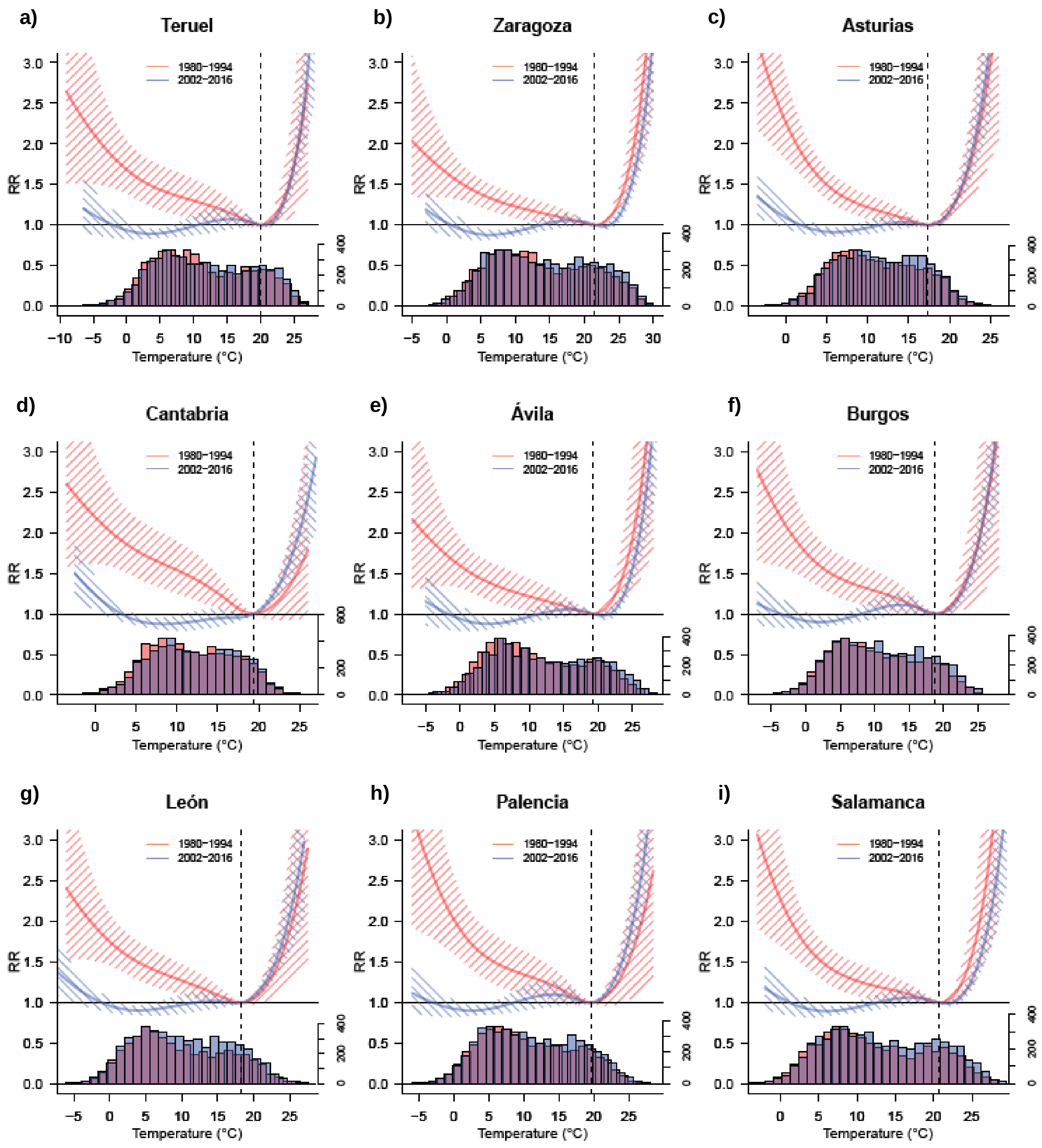

Supplementary Figure 6: **Relative risk of death from respiratory diseases in the Spanish provinces, with related daily temperature distributions.** The relative risk (RR) curves are estimated with the corresponding 95% empirical CI. The vertical dashed lines represent the Minimum Mortality Temperature (MMT) of the subperiod 1980-1994 is used as the centring temperature for the two subperiods (“*invariant centring temperature assumption*”, see Methods).

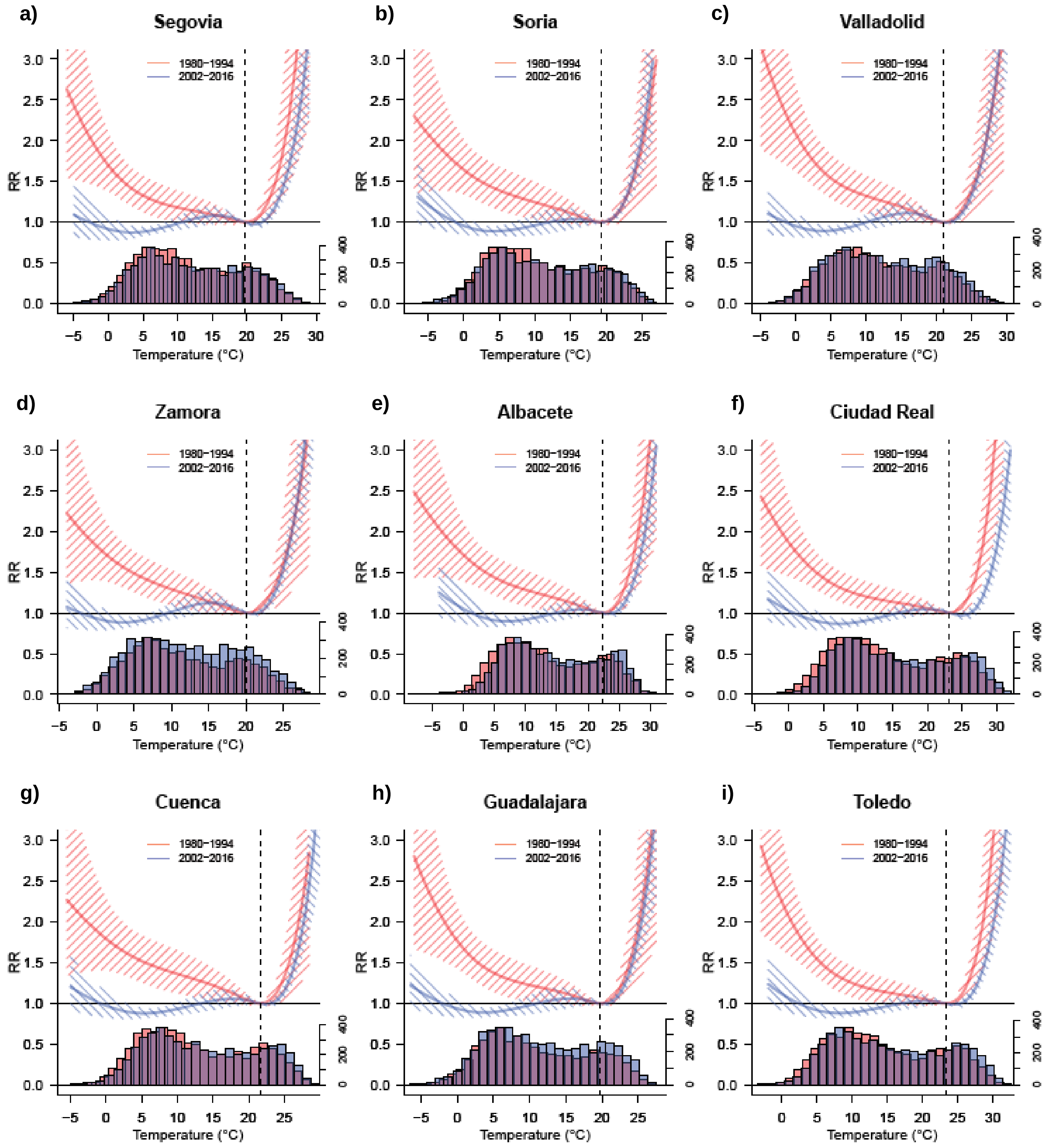

Supplementary Figure 7: **Relative risk of death from respiratory diseases in the Spanish provinces, with related daily temperature distributions.** The relative risk (RR) curves are estimated with the corresponding 95% empirical CI. The vertical dashed lines represent the Minimum Mortality Temperature (MMT) of the subperiod 1980-1994 is used as the centring temperature for the two subperiods (“*invariant centring temperature assumption*”, see Methods).

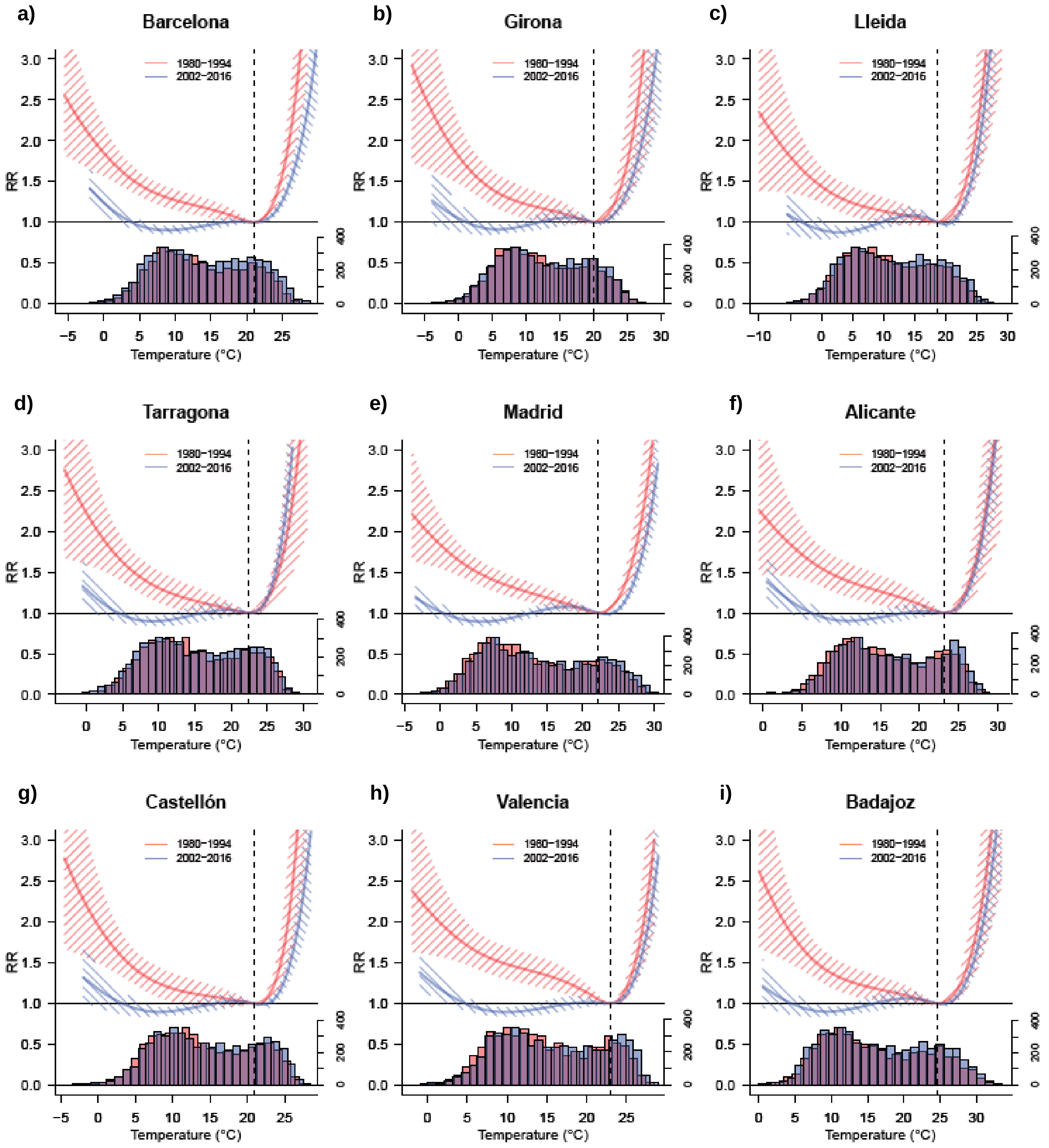

Supplementary Figure 8: **Relative risk of death from respiratory diseases in the Spanish provinces, with related daily temperature distributions.** The relative risk (RR) curves are estimated with the corresponding 95% empirical CI. The vertical dashed lines represent the Minimum Mortality Temperature (MMT) of the subperiod 1980-1994 is used as the centring temperature for the two subperiods (“*invariant centring temperature assumption*”, see Methods).

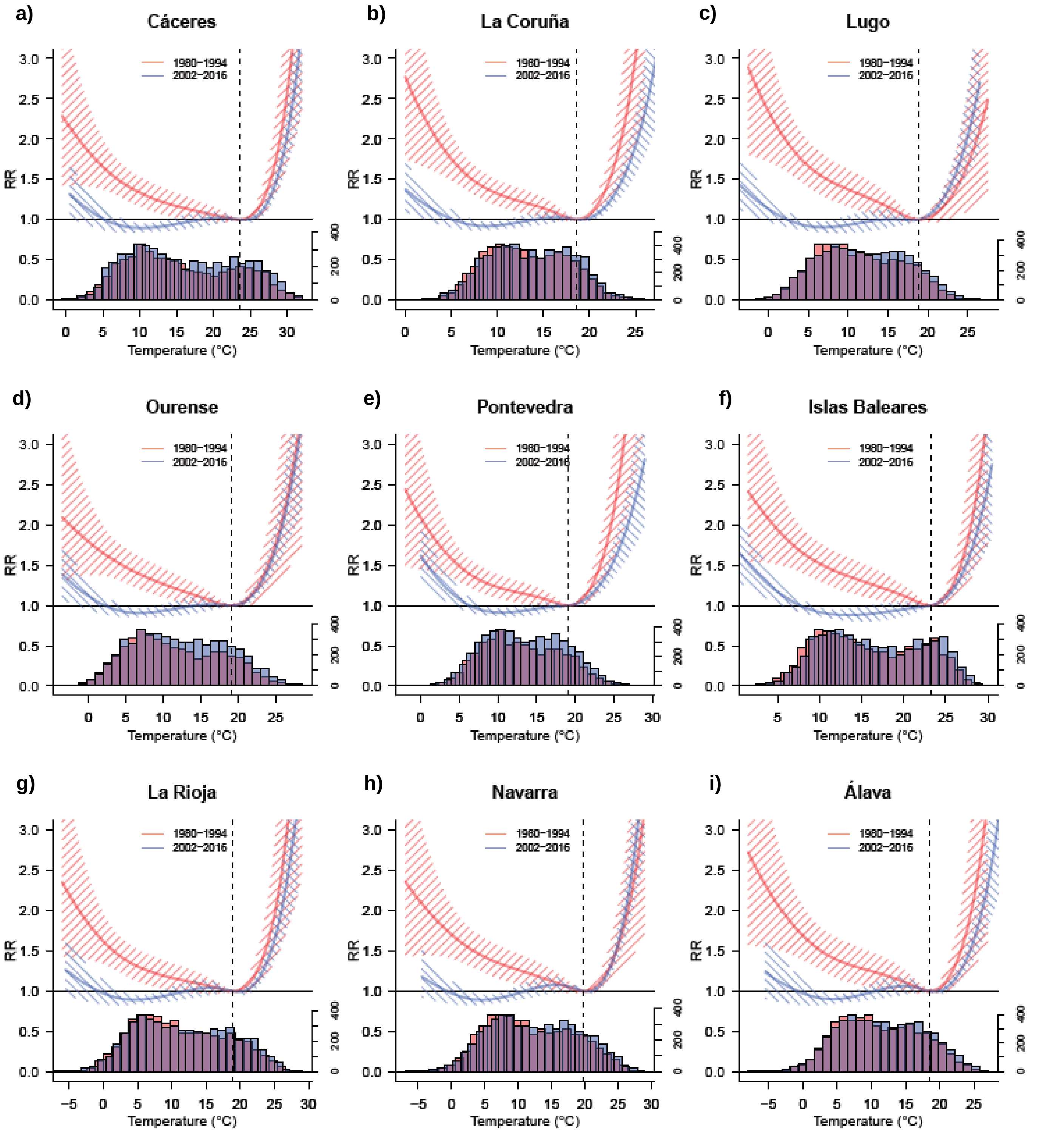

Supplementary Figure 9: **Relative risk of death from respiratory diseases in the Spanish provinces, with related daily temperature distributions.** The relative risk (RR) curves are estimated with the corresponding 95% empirical CI. The vertical dashed lines represent the Minimum Mortality Temperature (MMT) of the subperiod 1980-1994 is used as the centring temperature for the two subperiods (“*invariant centring temperature assumption*”, see Methods).

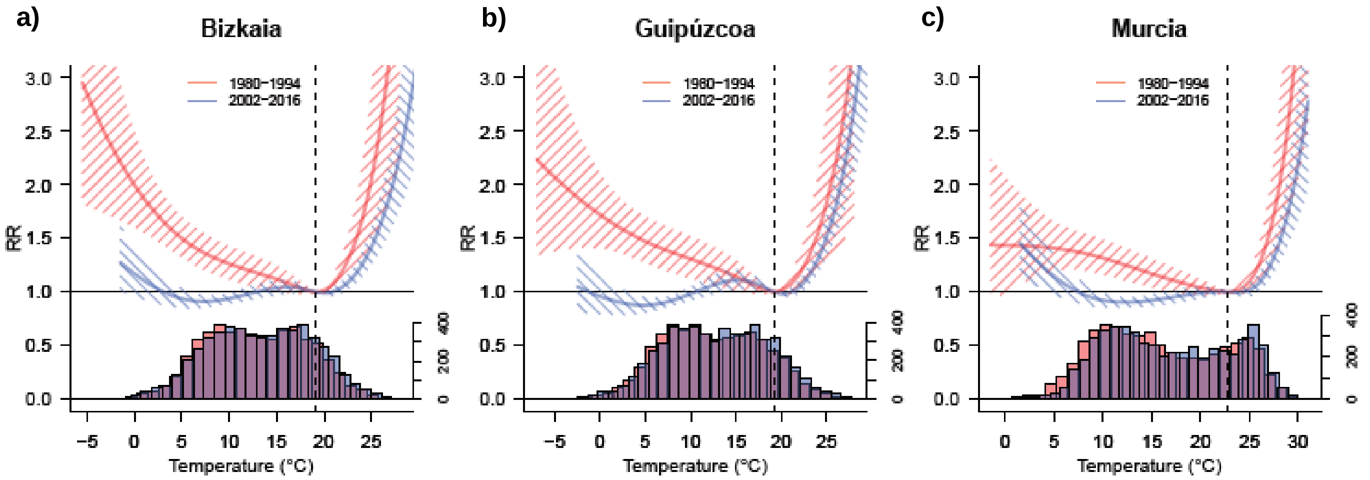

Supplementary Figure 10: **Relative risk of death from respiratory diseases in the Spanish provinces, with related daily temperature distributions.** The relative risk (RR) curves are estimated with the corresponding 95% empirical CI. The vertical dashed lines represent the Minimum Mortality Temperature (MMT) of the subperiod 1980-1994 is used as the centring temperature for the two subperiods ("*invariant centring temperature assumption*", see Methods).

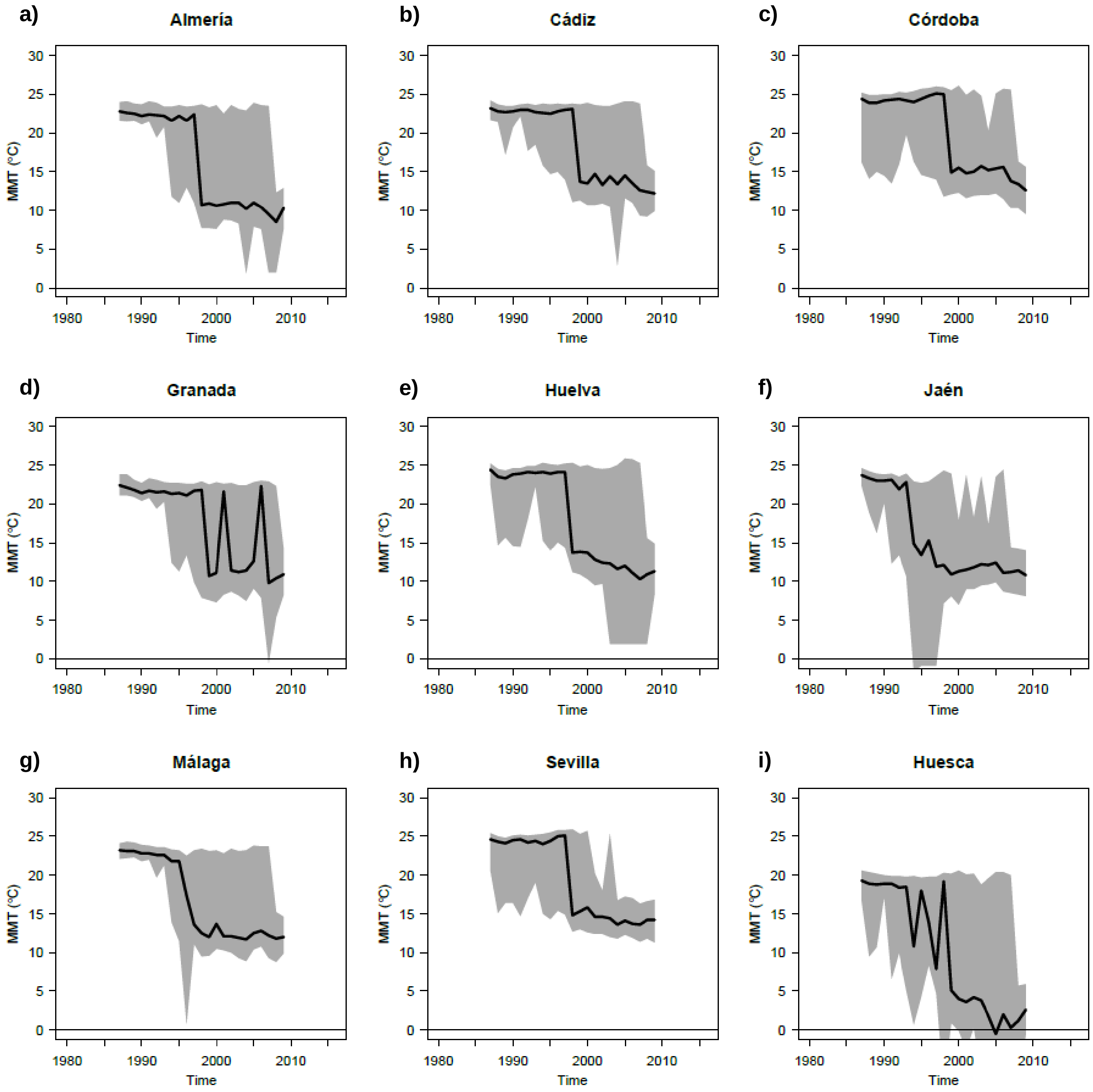

Supplementary Figure 11: **Evolution of the overall minimum mortality temperature (°C) by province.** MMT=minimum mortality temperature. The MMTs are estimated with the corresponding 95% empirical CI.

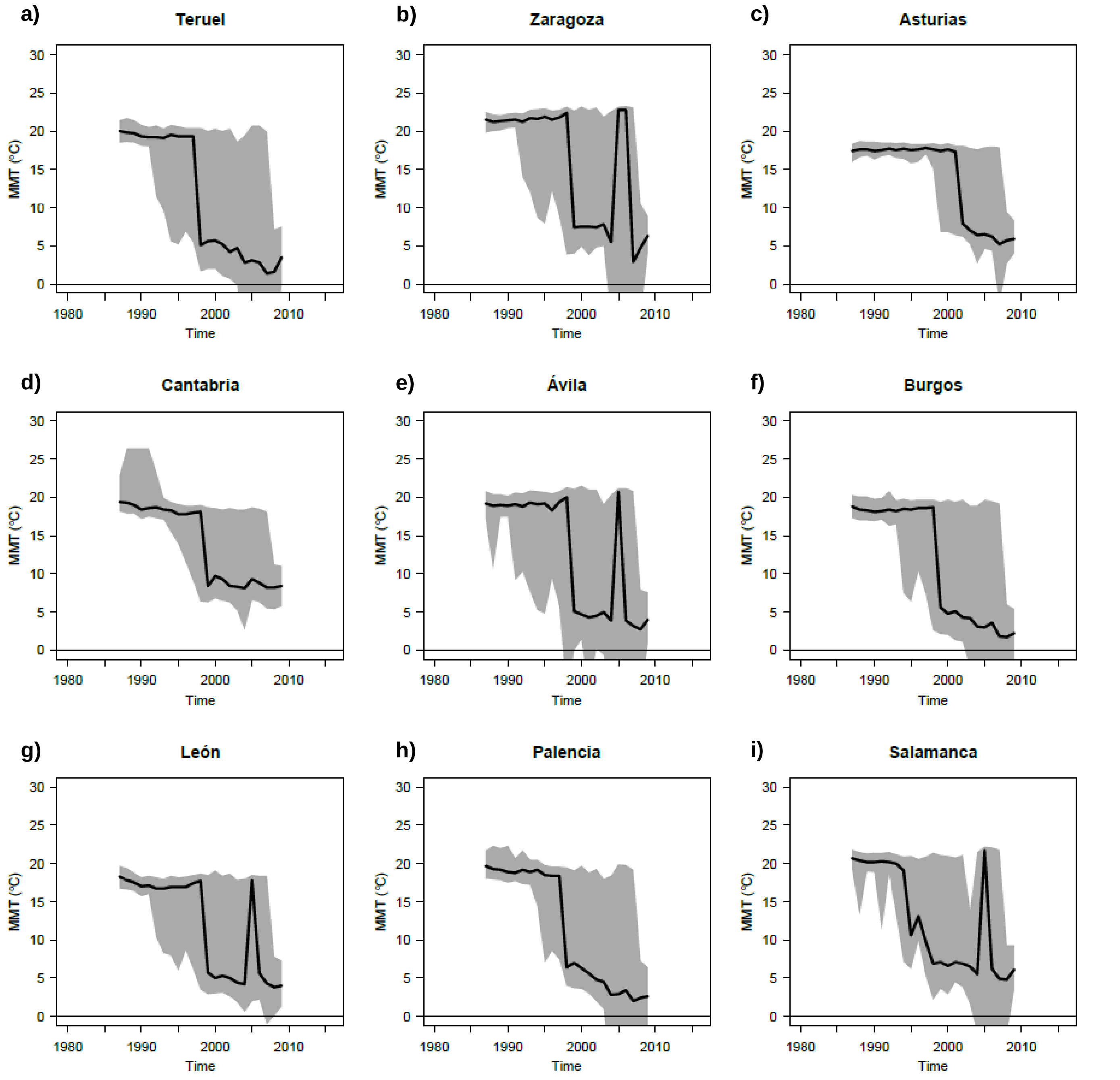

Supplementary Figure 12: **Evolution of the overall minimum mortality temperature (°C) by province.** MMT=minimum mortality temperature. The MMTs are estimated with the corresponding 95% empirical CI.

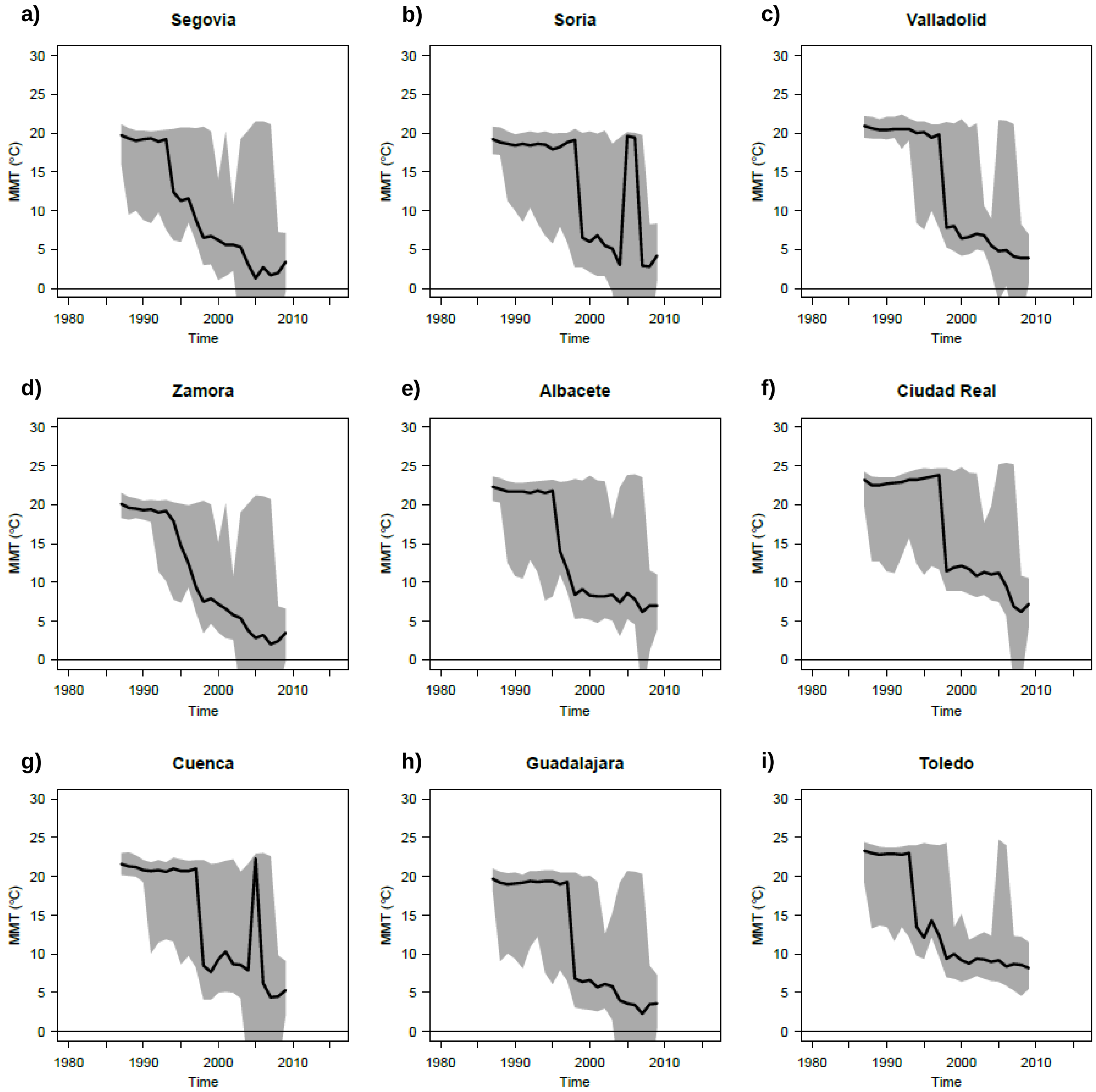

Supplementary Figure 13: **Evolution of the overall minimum mortality temperature (°C) by province.** MMT=minimum mortality temperature. The MMTs are estimated with the corresponding 95% empirical CI.

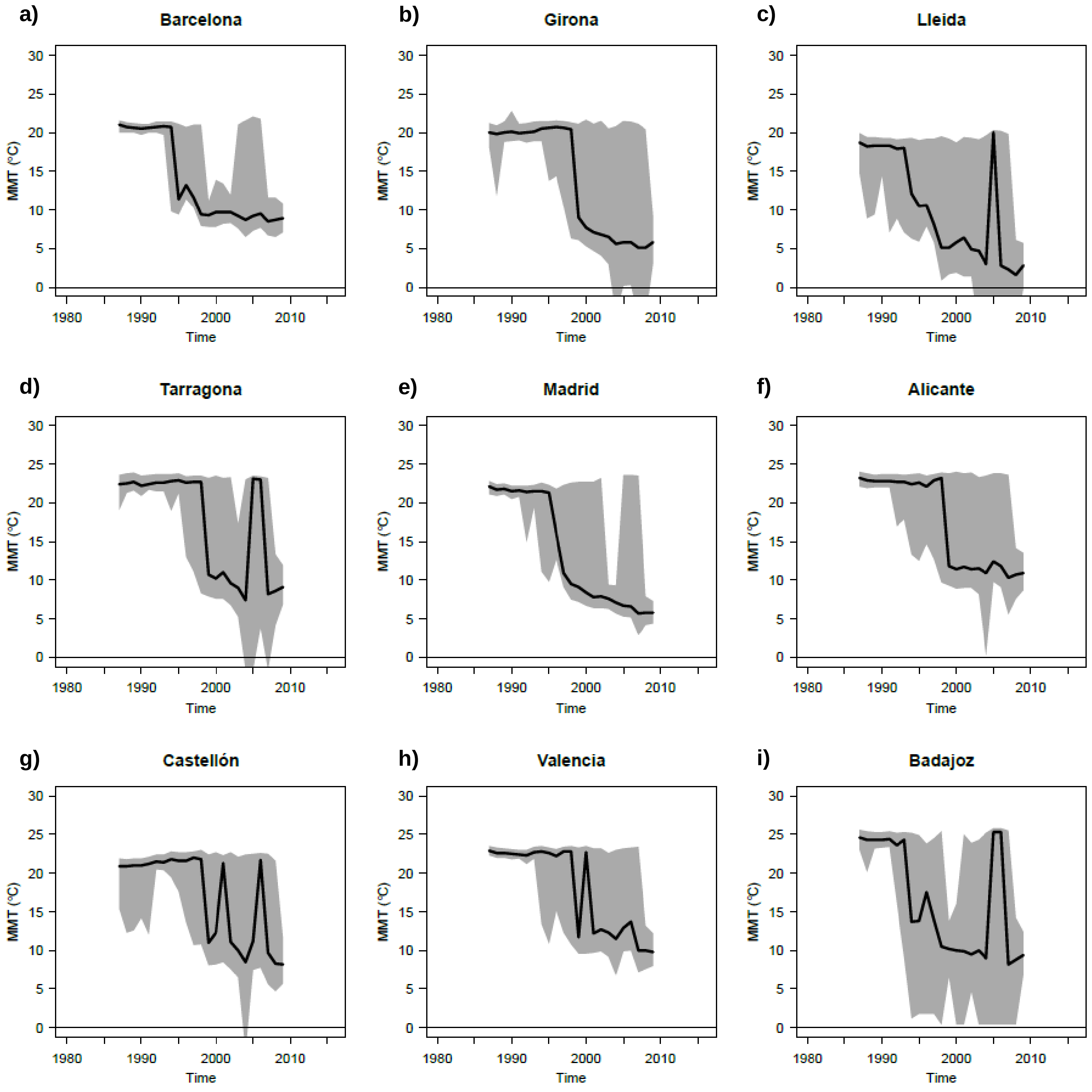

Supplementary Figure 14: **Evolution of the overall minimum mortality temperature (°C) by province.** MMT=minimum mortality temperature. The MMTs are estimated with the corresponding 95% empirical CI.

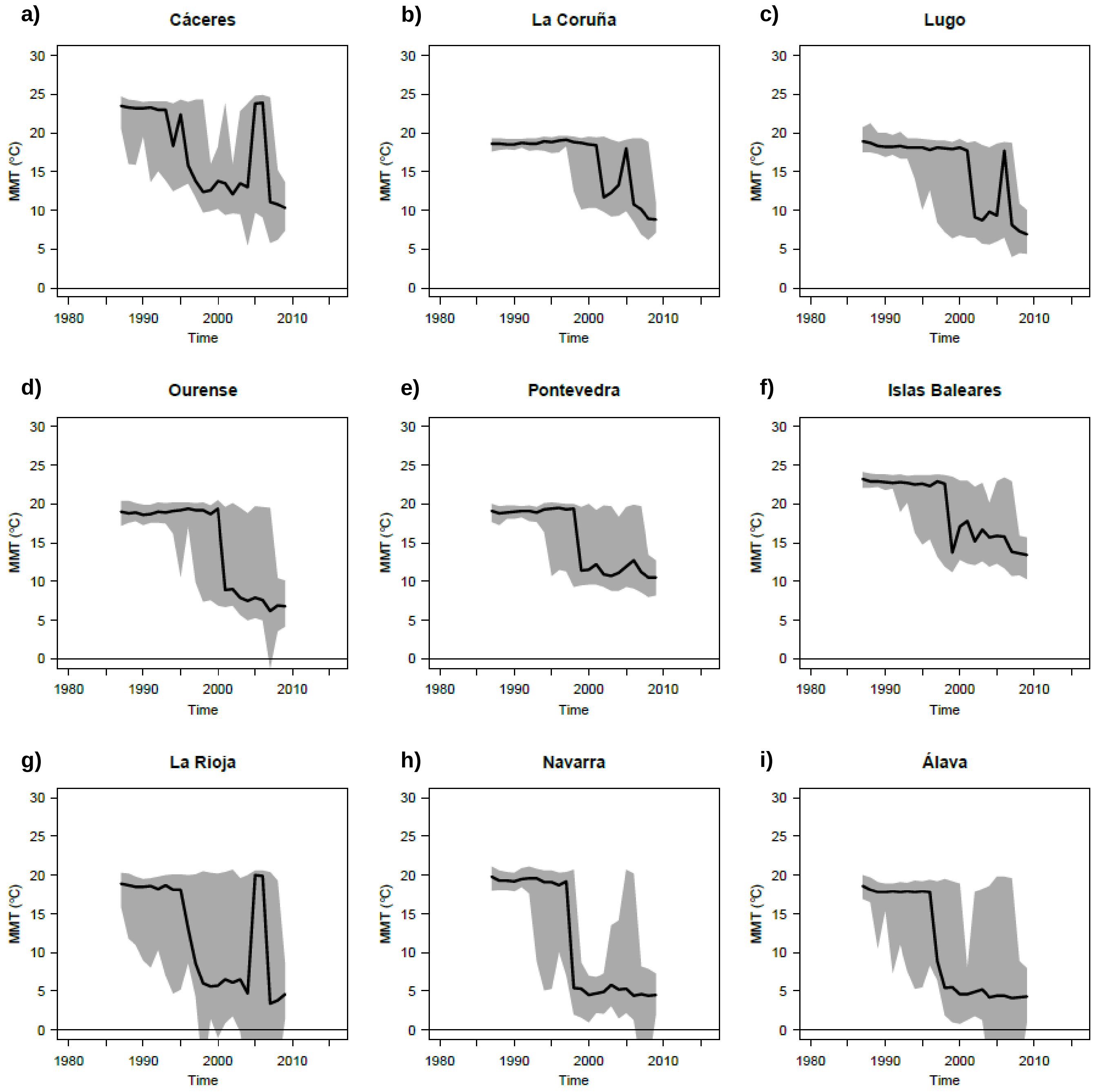

Supplementary Figure 15: **Evolution of the overall minimum mortality temperature (°C) by province.** MMT=minimum mortality temperature. The MMTs are estimated with the corresponding 95% empirical CI.

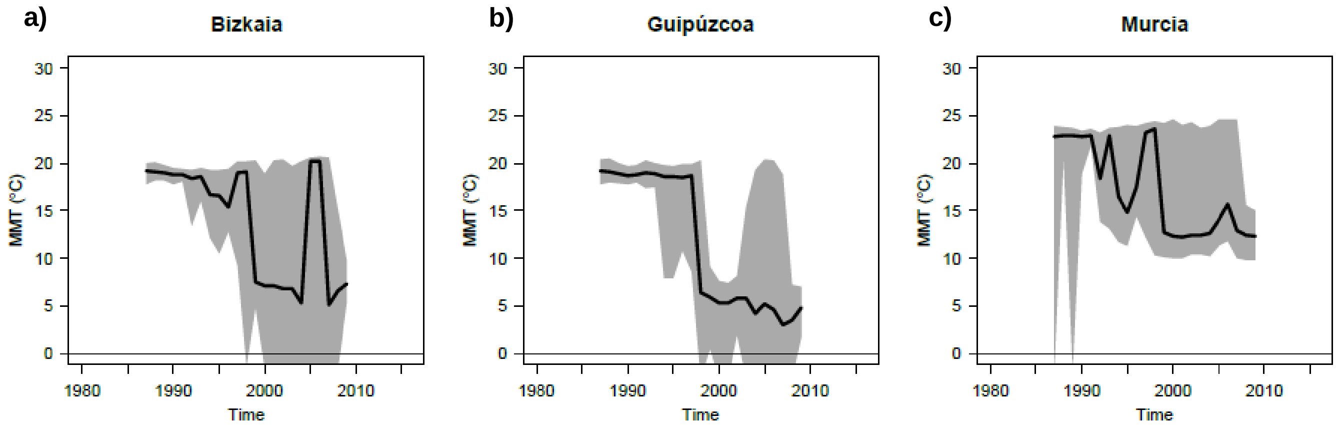

Supplementary Figure 16: **Evolution of the overall minimum mortality temperature (°C) by province.** MMT=minimum mortality temperature. The MMTs are estimated with the corresponding 95% empirical CI.

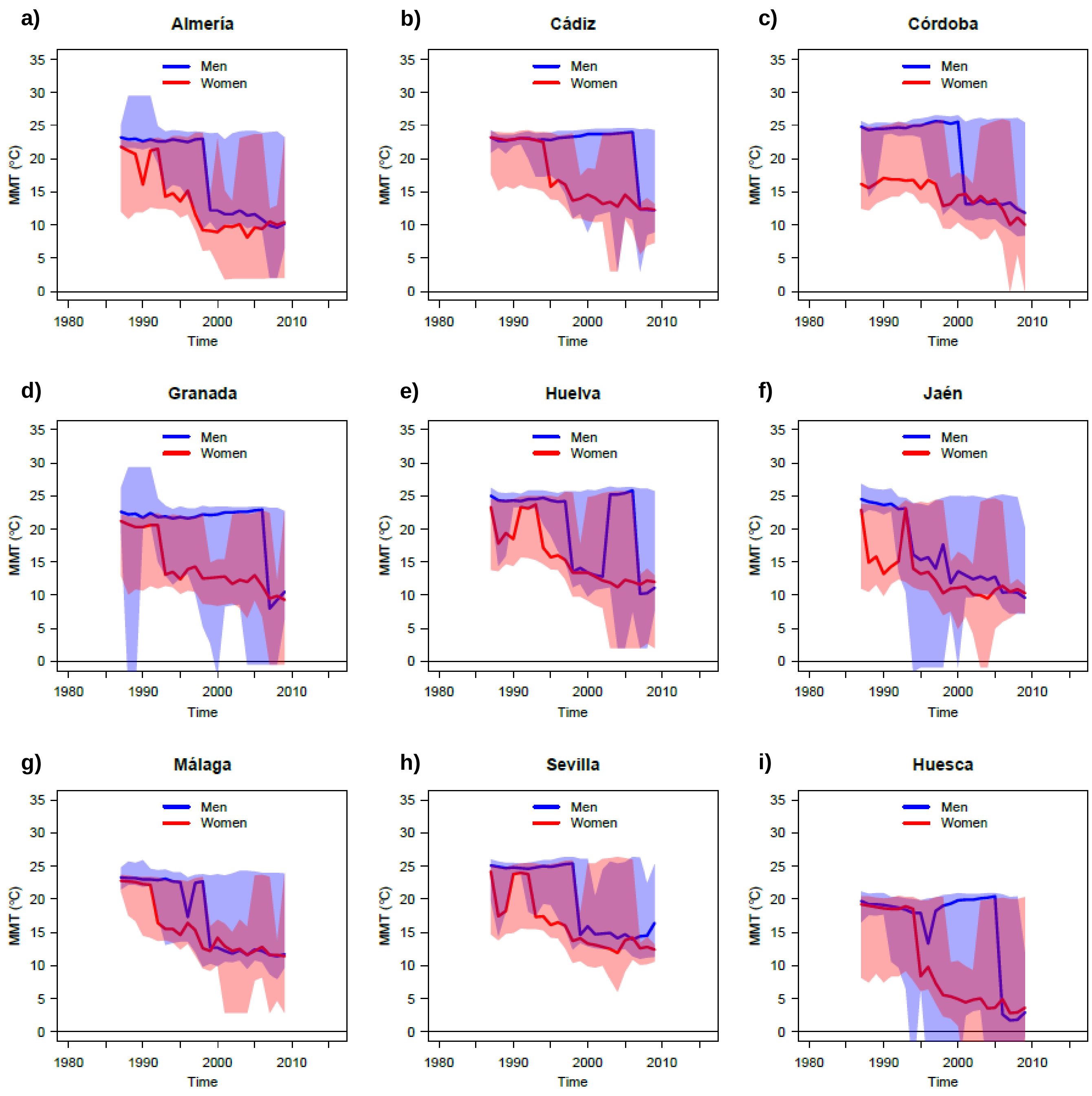

Supplementary Figure 17: **Evolution of the minimum mortality temperature (°C) by sex and province.** MMT=minimum mortality temperature. The MMTs are estimated with the corresponding 95% empirical CI.

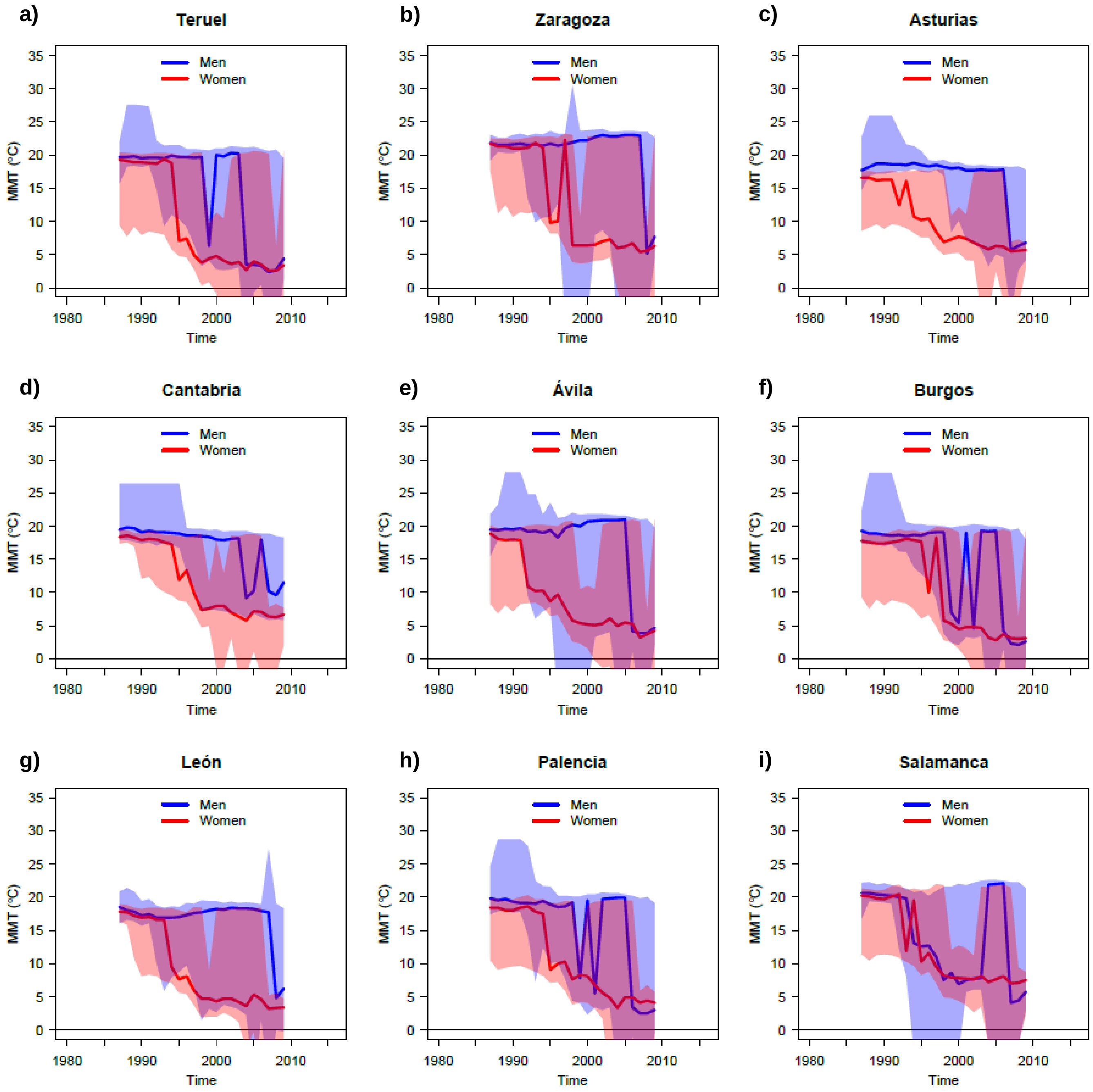

Supplementary Figure 18: **Evolution of the minimum mortality temperature (°C) by sex and province.** MMT=minimum mortality temperature. The MMTs are estimated with the corresponding 95% empirical CI.

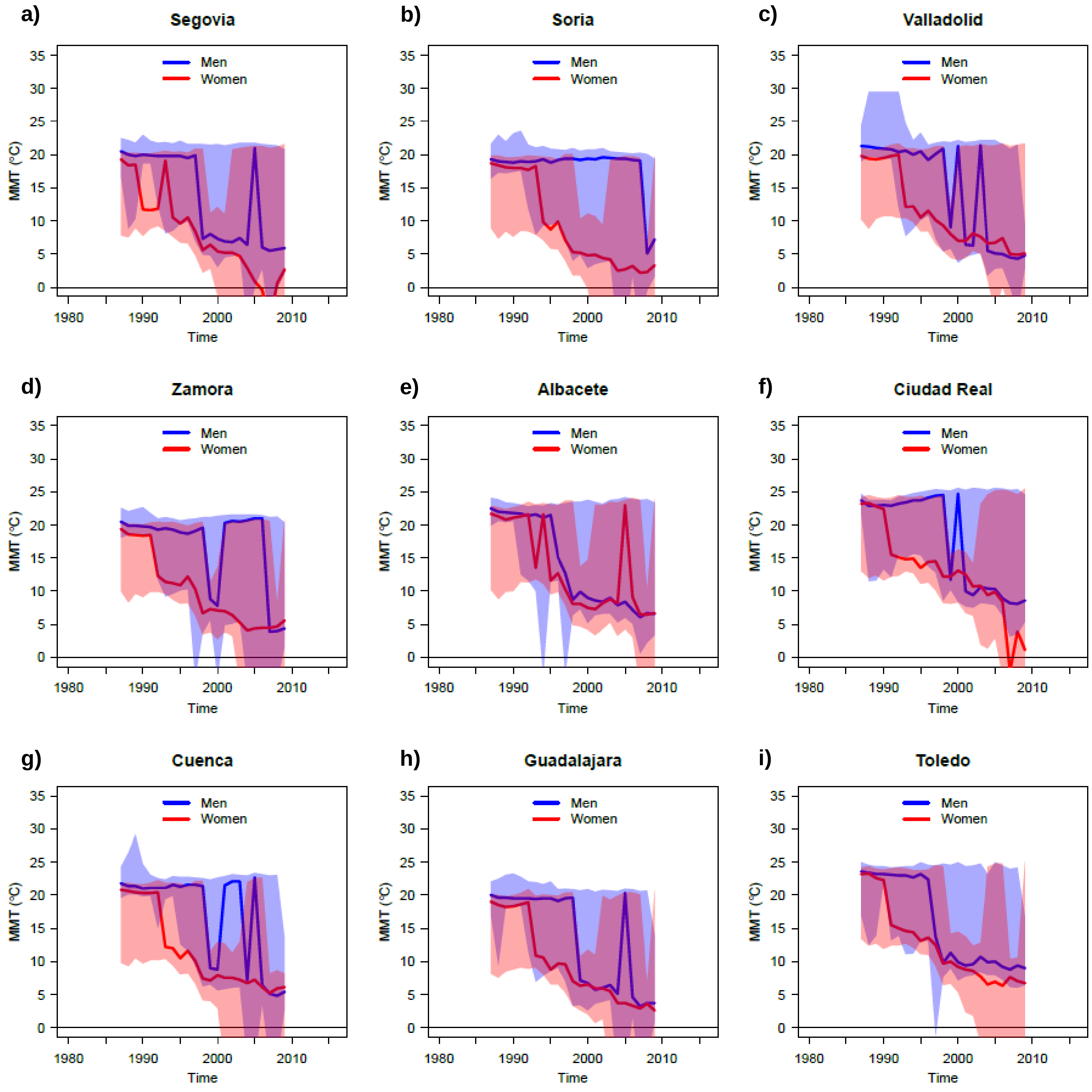

Supplementary Figure 19: **Evolution of the minimum mortality temperature (°C) by sex and province.** MMT=minimum mortality temperature. The MMTs are estimated with the corresponding 95% empirical CI.

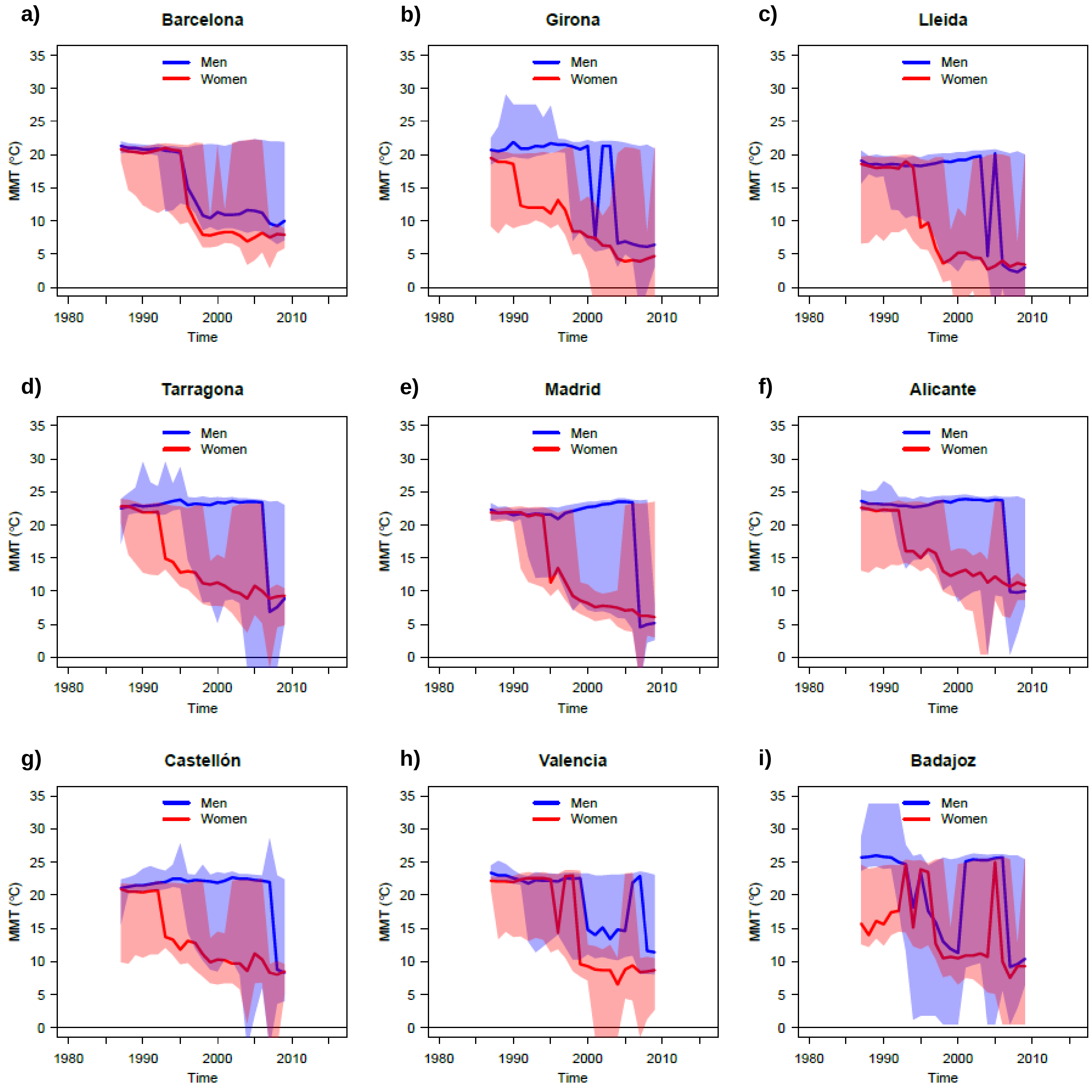

Supplementary Figure 20: **Evolution of the minimum mortality temperature (°C) by sex and province.** MMT=minimum mortality temperature. The MMTs are estimated with the corresponding 95% empirical CI.

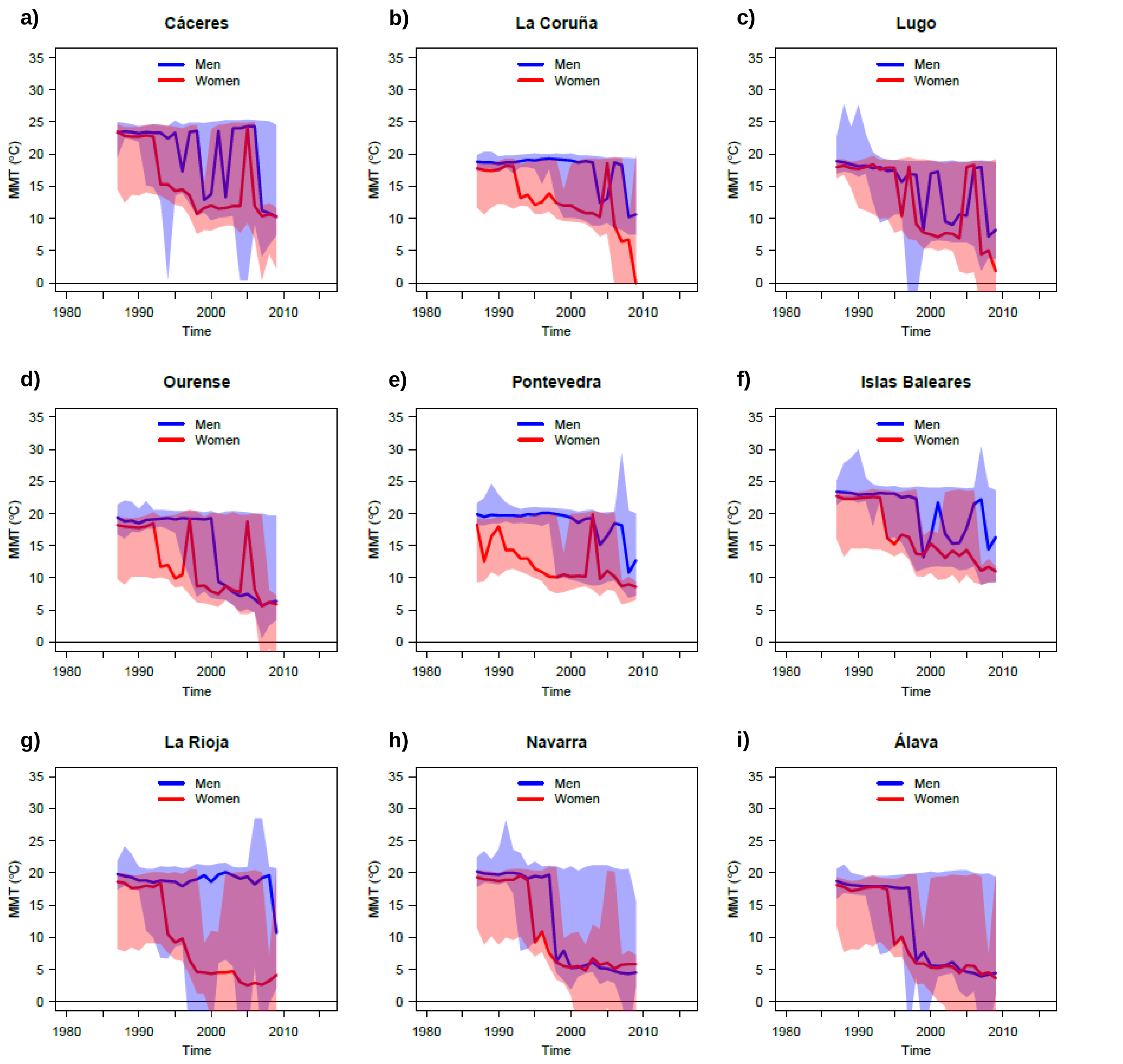

Supplementary Figure 21: **Evolution of the minimum mortality temperature (°C) by sex and province.** MMT=minimum mortality temperature. The MMTs are estimated with the corresponding 95% empirical CI.

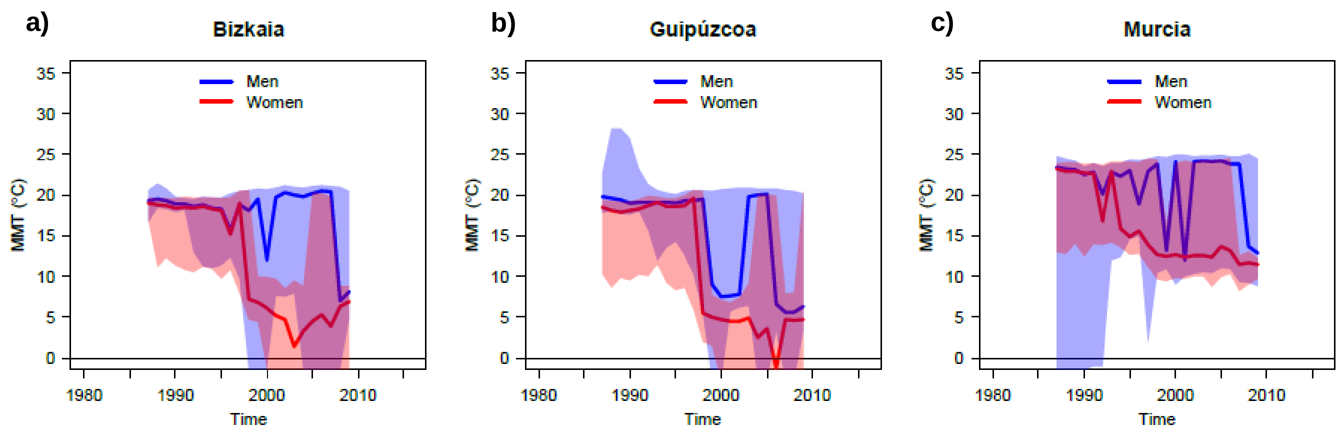

Supplementary Figure 22: **Evolution of the minimum mortality temperature (°C) by sex and province.** MMT=minimum mortality temperature. The MMTs are estimated with the corresponding 95% empirical CI.

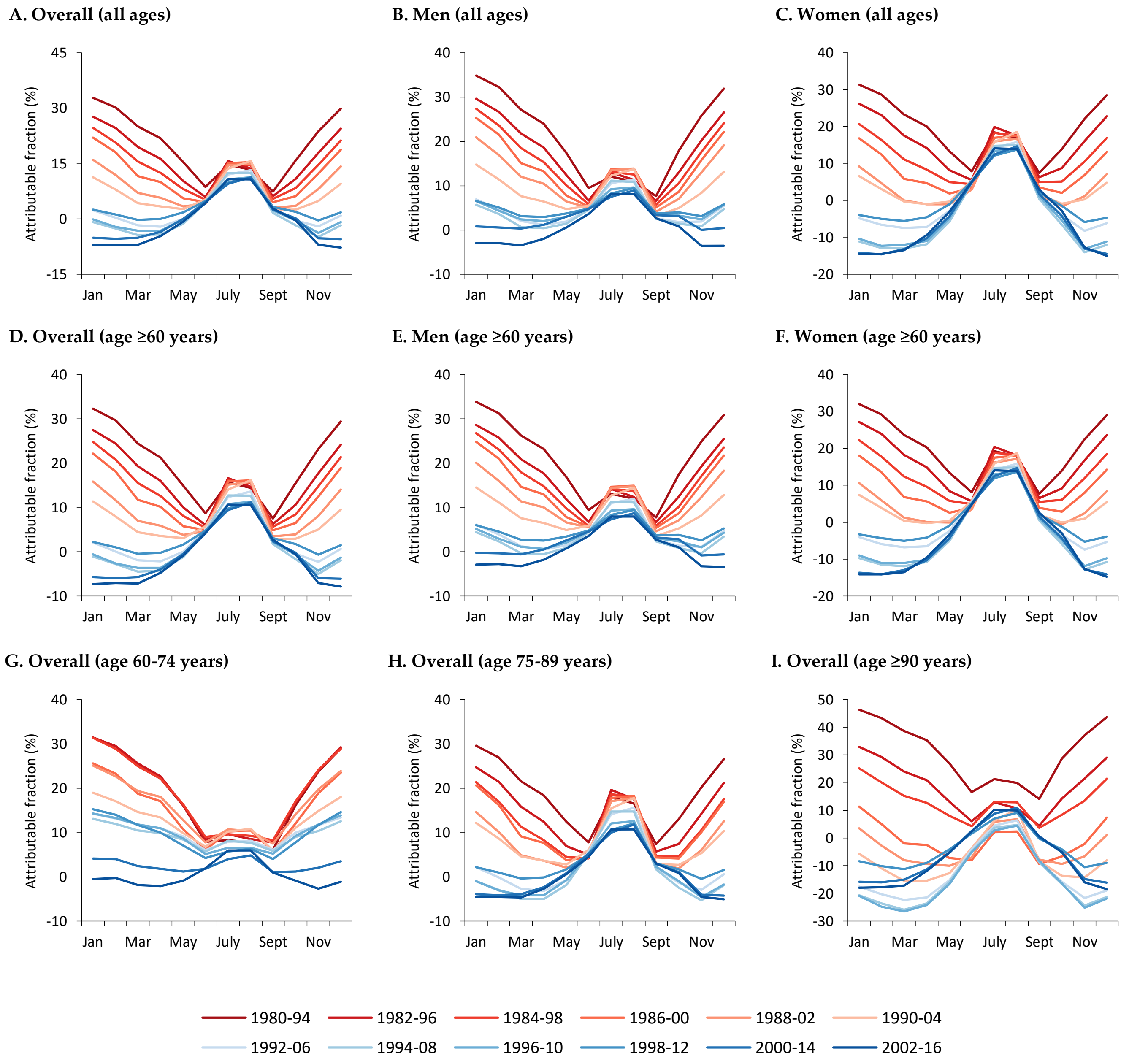

Supplementary Figure 23: **Monthly Attributable Fraction of respiratory disease mortality in Spain by sex and age for 15-year subperiods.** The Minimum Mortality Temperature (MMT) of the subperiod 1980-1994 is used as the centring temperature for the two subperiods (“*invariant centring temperature assumption*”, see Methods). AF=Attributable Fraction.

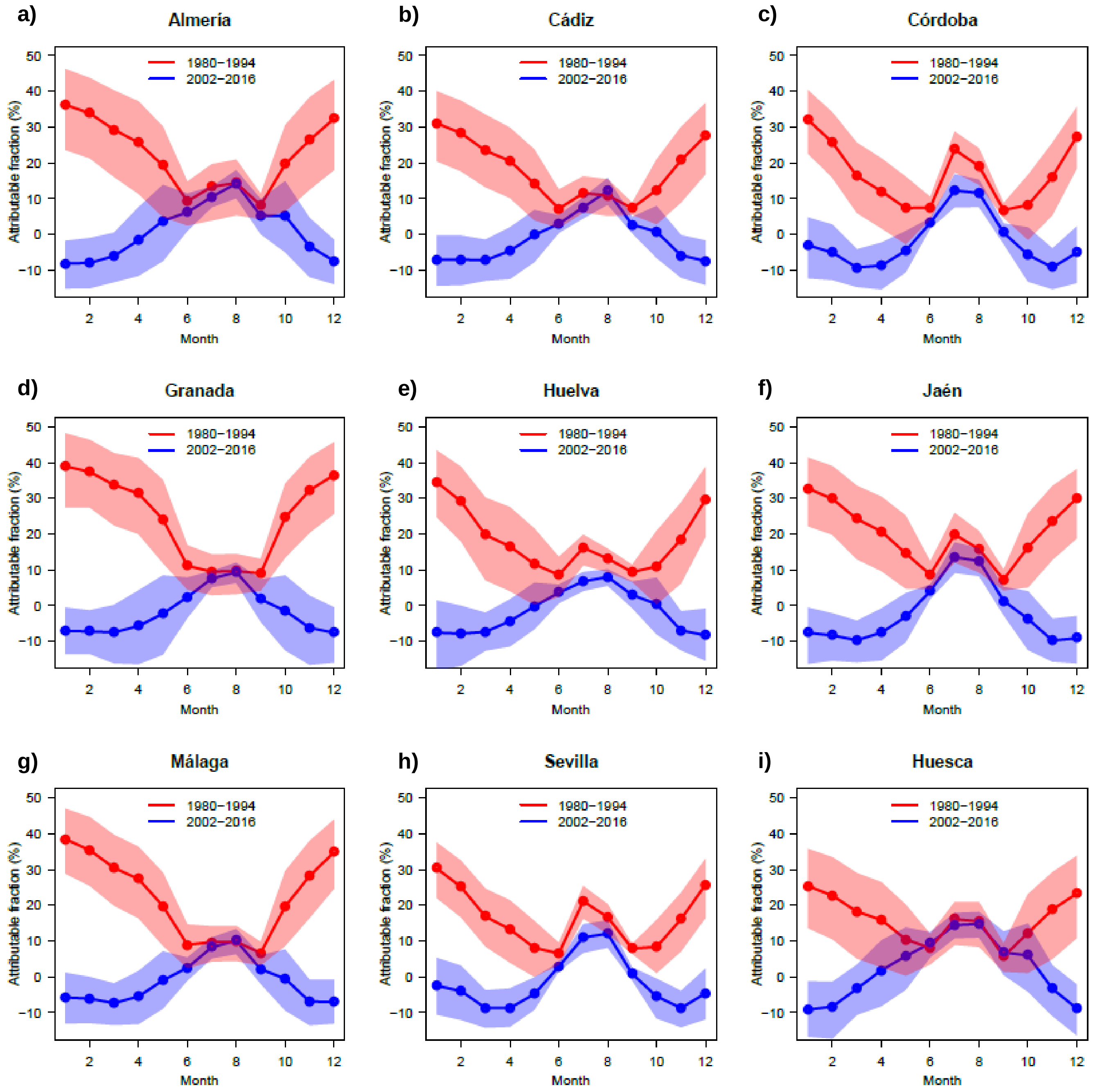

Supplementary Figure 24: **Monthly Attributable Fraction of respiratory disease mortality in the Spanish provinces.** The attributable fractions (AF) are estimated with the corresponding 95% empirical CI. The Minimum Mortality Temperature (MMT) of the subperiod 1980-1994 is used as the centring temperature for the two subperiods (*"invariant centring temperature assumption"*, see Methods).

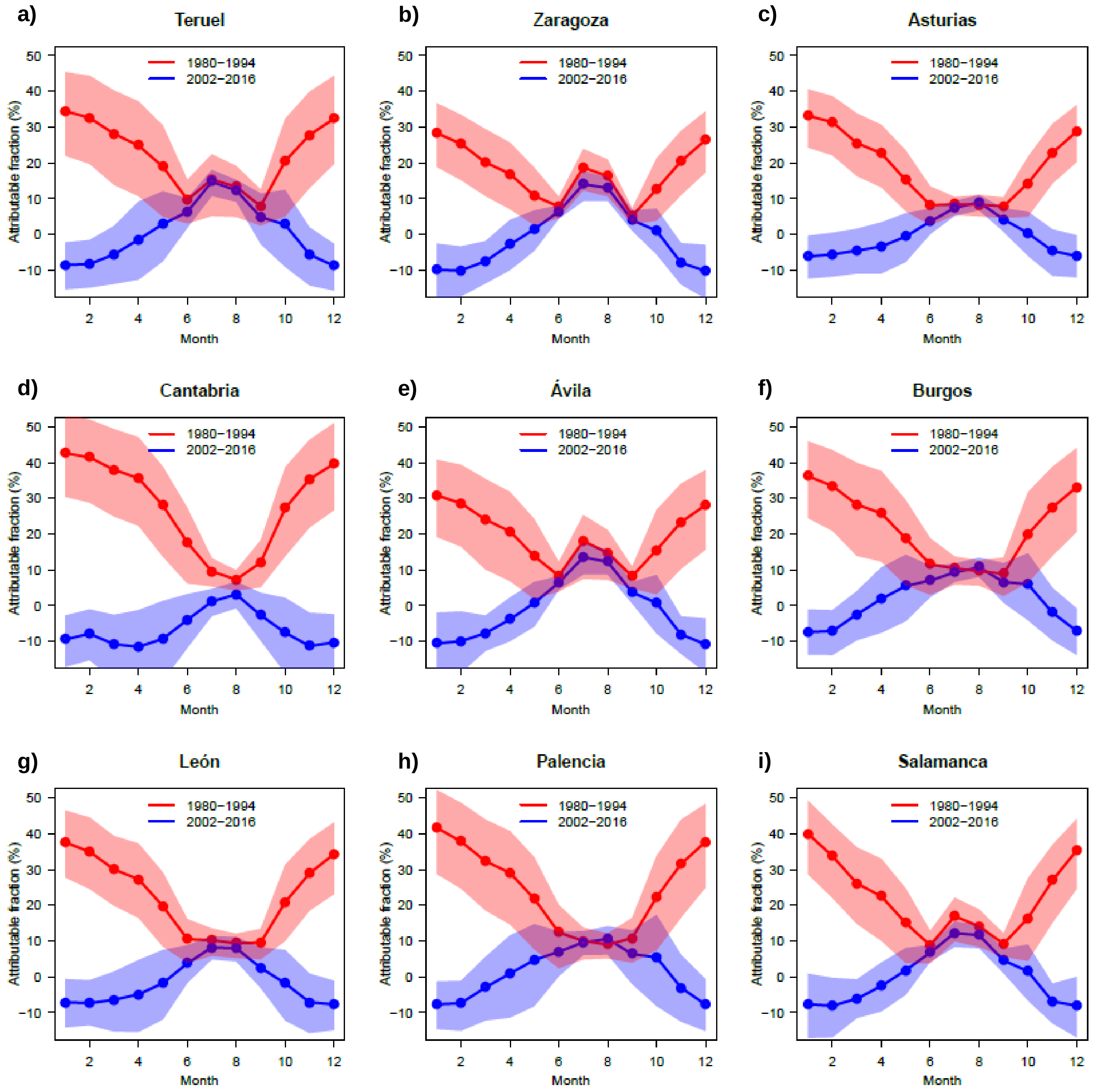

Supplementary Figure 25: **Monthly Attributable Fraction of respiratory disease mortality in the Spanish provinces.** The attributable fractions (AF) are estimated with the corresponding 95% empirical CI. The Minimum Mortality Temperature (MMT) of the subperiod 1980-1994 is used as the centring temperature for the two subperiods (*"invariant centring temperature assumption"*, see Methods).

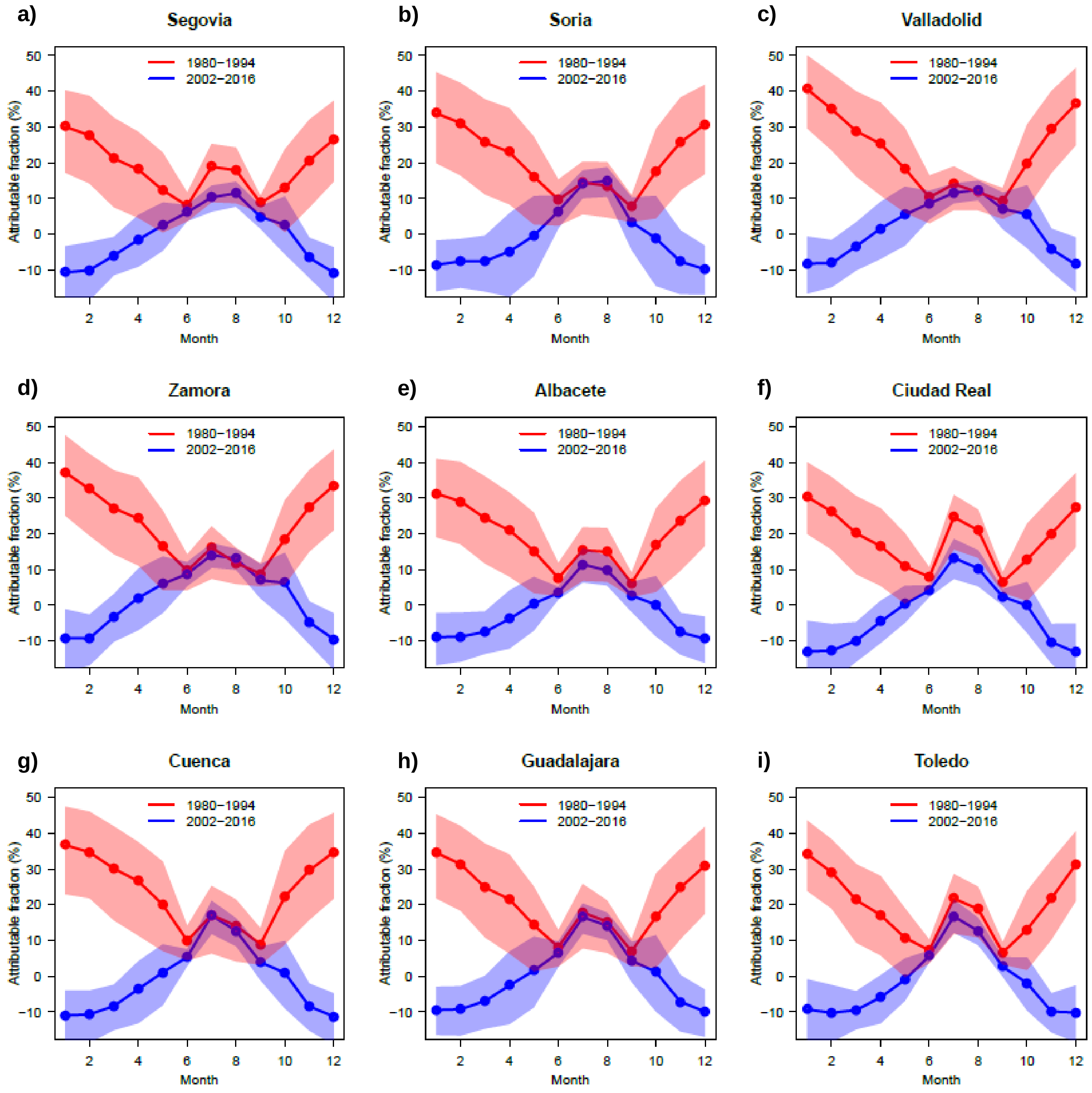

Supplementary Figure 26: **Monthly Attributable Fraction of respiratory disease mortality in the Spanish provinces.** The attributable fractions (AF) are estimated with the corresponding 95% empirical CI. The Minimum Mortality Temperature (MMT) of the subperiod 1980-1994 is used as the centring temperature for the two subperiods ("*invariant centring temperature assumption*", see Methods).

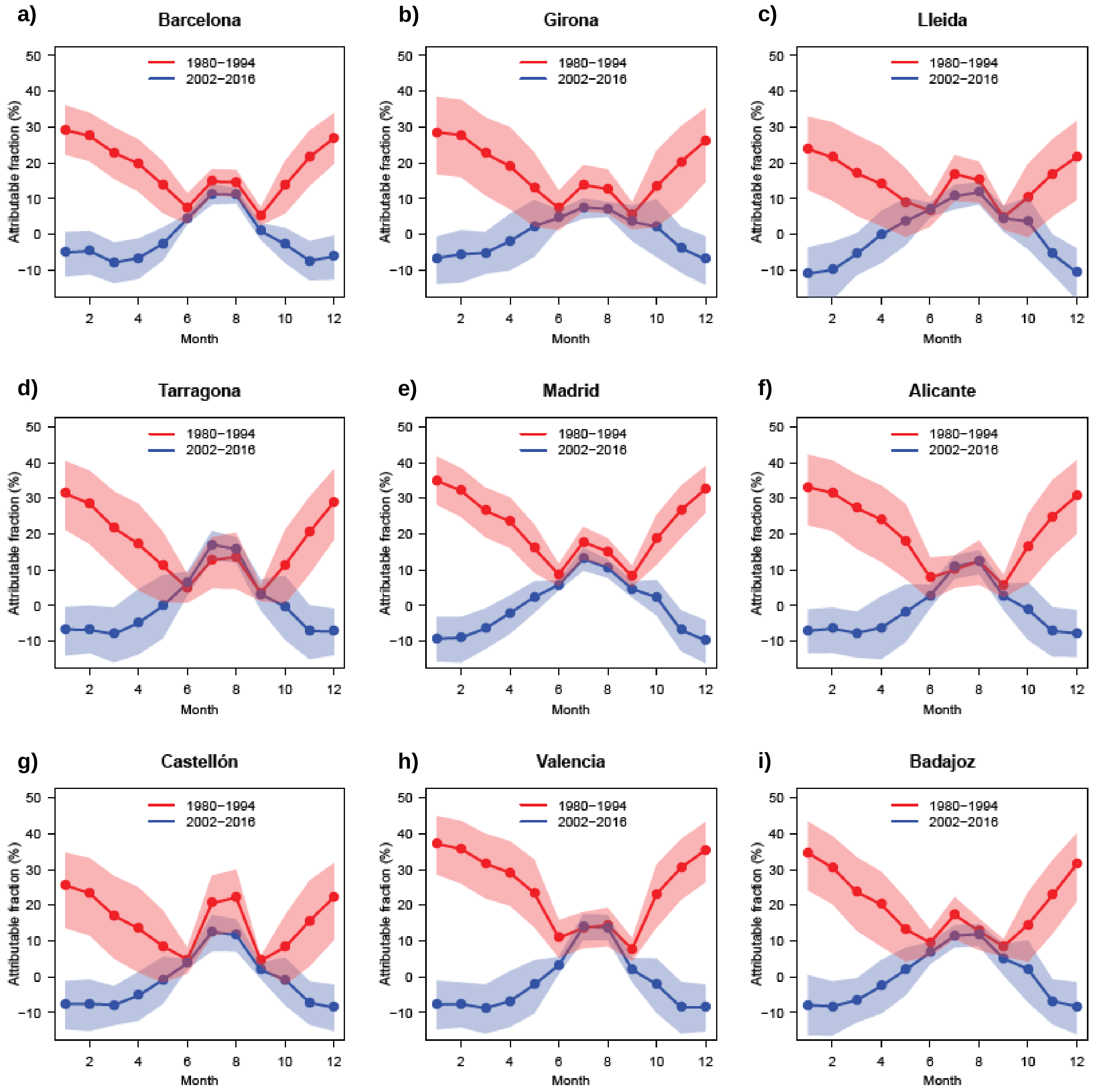

Supplementary Figure 27: **Monthly Attributable Fraction of respiratory disease mortality in the Spanish provinces.** The attributable fractions (AF) are estimated with the corresponding 95% empirical CI. The Minimum Mortality Temperature (MMT) of the subperiod 1980-1994 is used as the centring temperature for the two subperiods (*"invariant centring temperature assumption"*, see Methods).

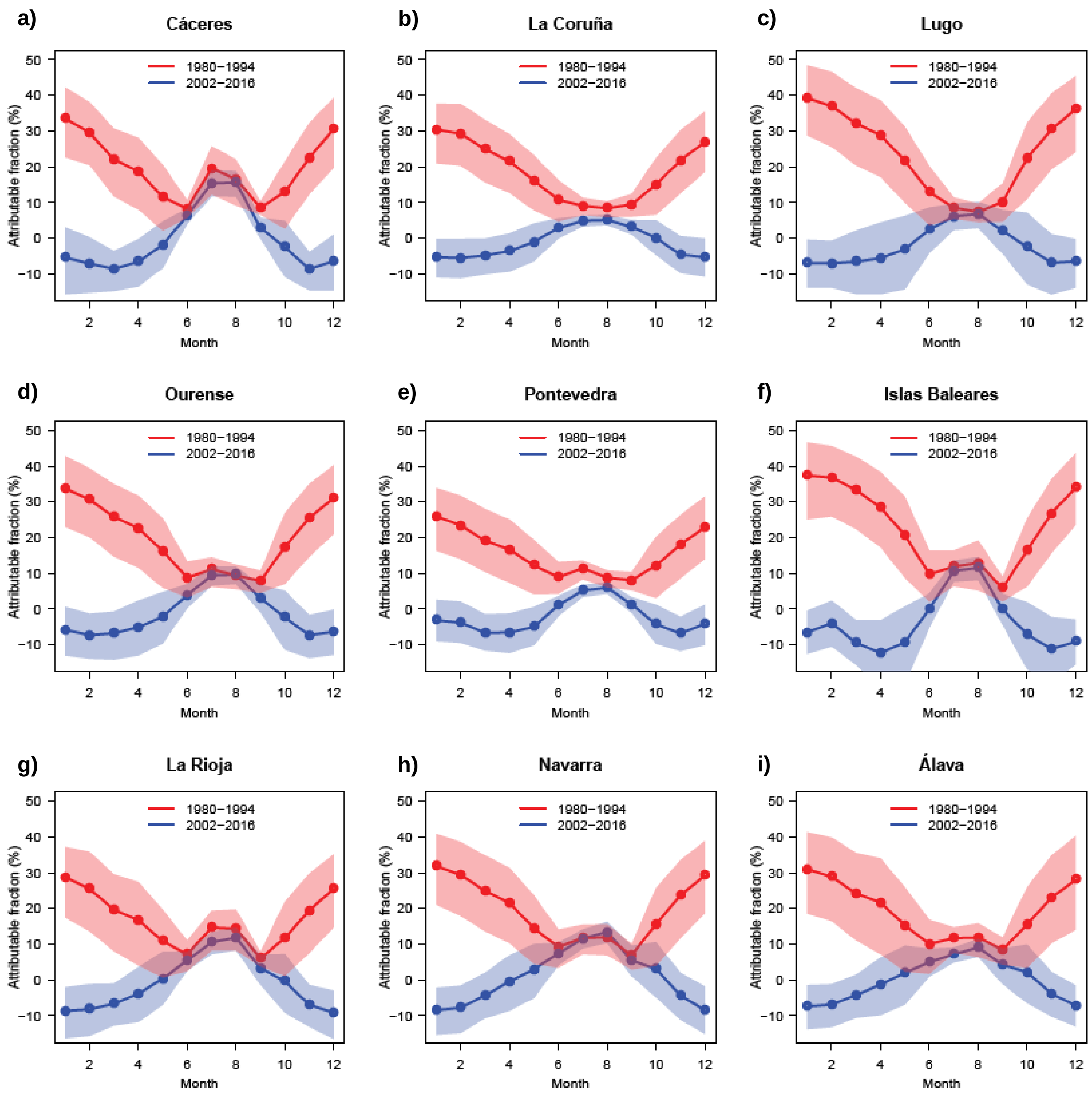

Supplementary Figure 28: **Monthly Attributable Fraction of respiratory disease mortality in the Spanish provinces.** The attributable fractions (AF) are estimated with the corresponding 95% empirical CI. The Minimum Mortality Temperature (MMT) of the subperiod 1980-1994 is used as the centring temperature for the two subperiods (*"invariant centring temperature assumption"*, see Methods).

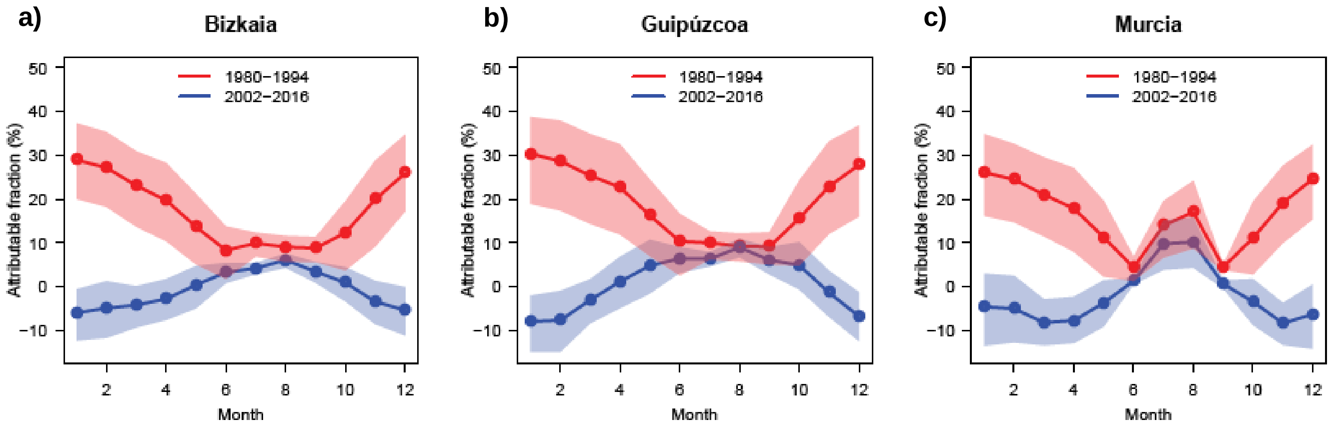

Supplementary Figure 29: **Monthly Attributable Fraction of respiratory disease mortality in the Spanish provinces.** The attributable fractions (AF) are estimated with the corresponding 95% empirical CI. The Minimum Mortality Temperature (MMT) of the subperiod 1980-1994 is used as the centring temperature for the two subperiods ("*invariant centring temperature assumption*", see Methods).

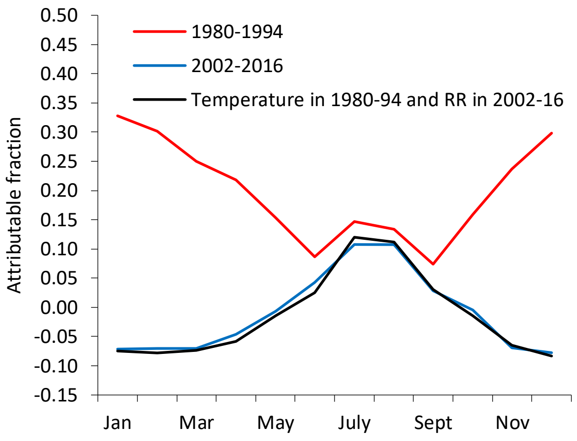

Supplementary Figure 30: **Monthly Attributable Fraction of respiratory disease mortality in Spain.** The Minimum Mortality Temperature (MMT) of the subperiod 1980-1994 is used as the centring temperature for the two subperiods ("*invariant centring temperature assumption*", see Methods). This figure shows values for the first (1980-1994, red curve) and last (2002-2016, blue curve) 15-year subperiods of the series. The black curve corresponds to estimations resulting from applying the daily time series of temperatures in 1980-1994 to the RR curves in 2002-2016. The small difference between the black and blue curves therefore shows the rather small effect of the warming, while the large difference between the black and red curves correspond to the dominant contribution of the changes in the RR.

A. Overall (all ages)

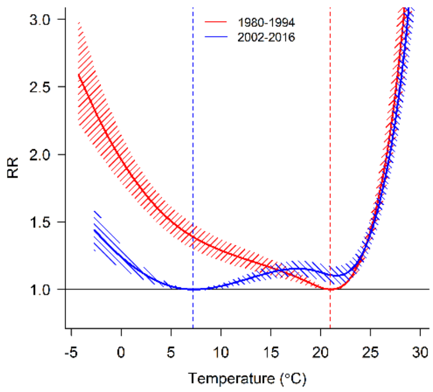

B. Men (all ages)

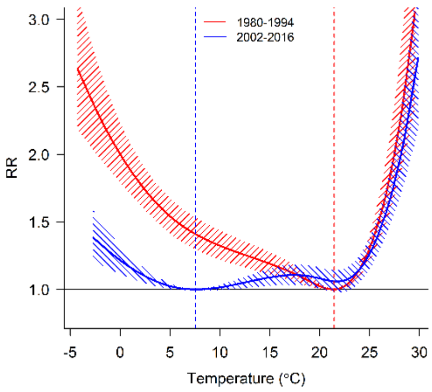

C. Women (all ages)

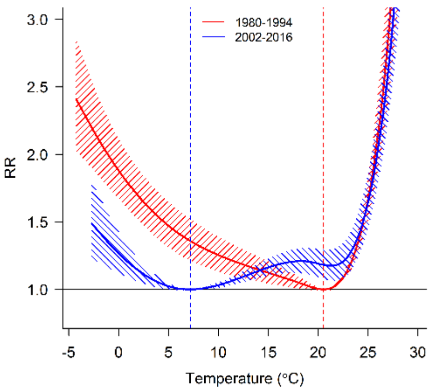

D. Overall (age ≥60 years)

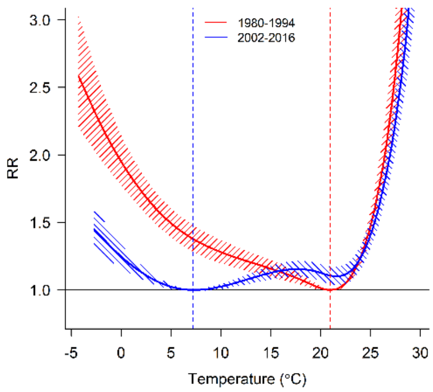

E. Men (age ≥60 years)

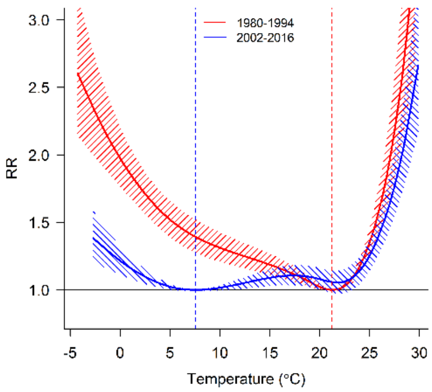

F. Women (age ≥60 years)

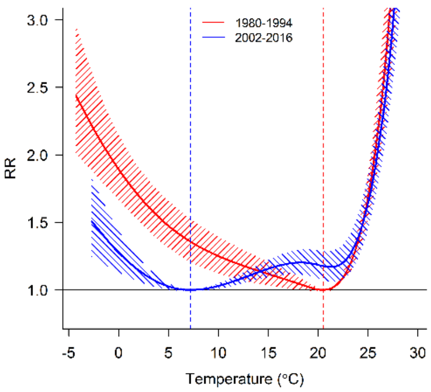

G. Overall (age 60-74 years)

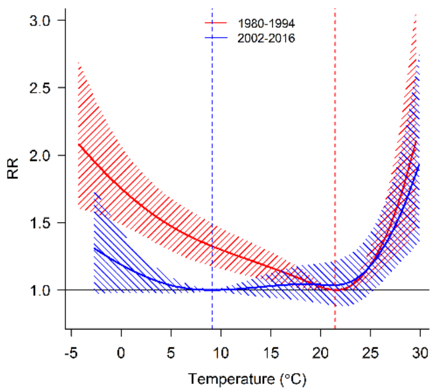

H. Overall (age 75-89 years)

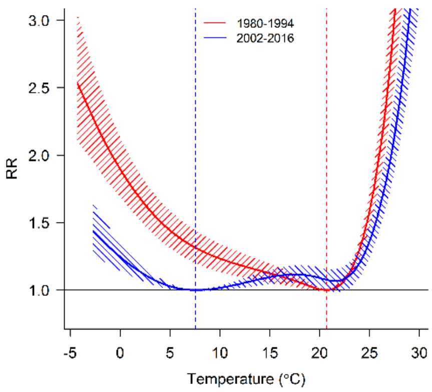

I. Overall (age ≥90 years)

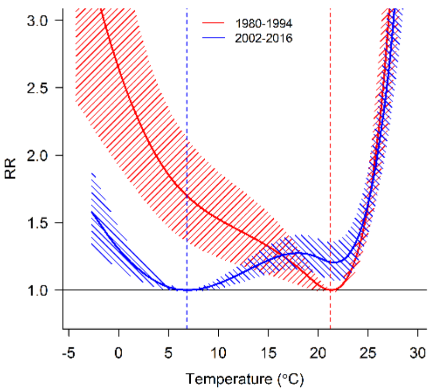

Supplementary Figure 31: **Relative risk of death from respiratory diseases in Spain for the periods 1980-1994 and 2002-2016.** The relative risk (RR) curves are estimated with the corresponding 95% empirical CI. Vertical dashed lines are minimum mortality temperatures (MMTs). The Minimum Mortality Temperature (MMT) of a given subperiod is used as the centring temperature of the subperiod (“*varying centring temperature assumption*”, see Methods).

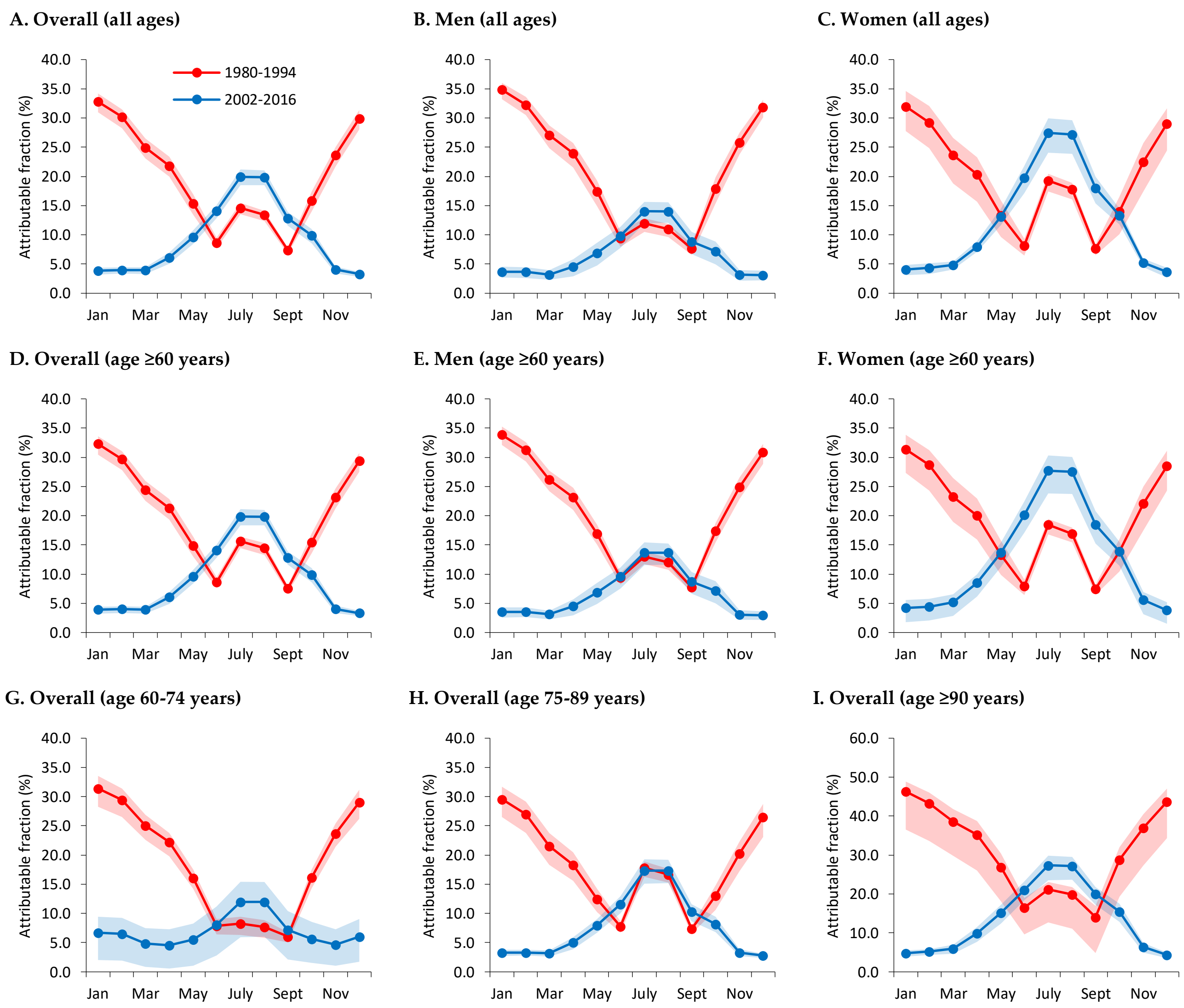

Supplementary Figure 32: **Monthly Attributable Fraction of respiratory disease mortality in Spain by sex and age.** The attributable fractions (AF) are estimated with the corresponding 95% empirical CI. The Minimum Mortality Temperature (MMT) of a given subperiod is used as the centring temperature of the subperiod ("*varying centring temperature assumption*", see Methods).

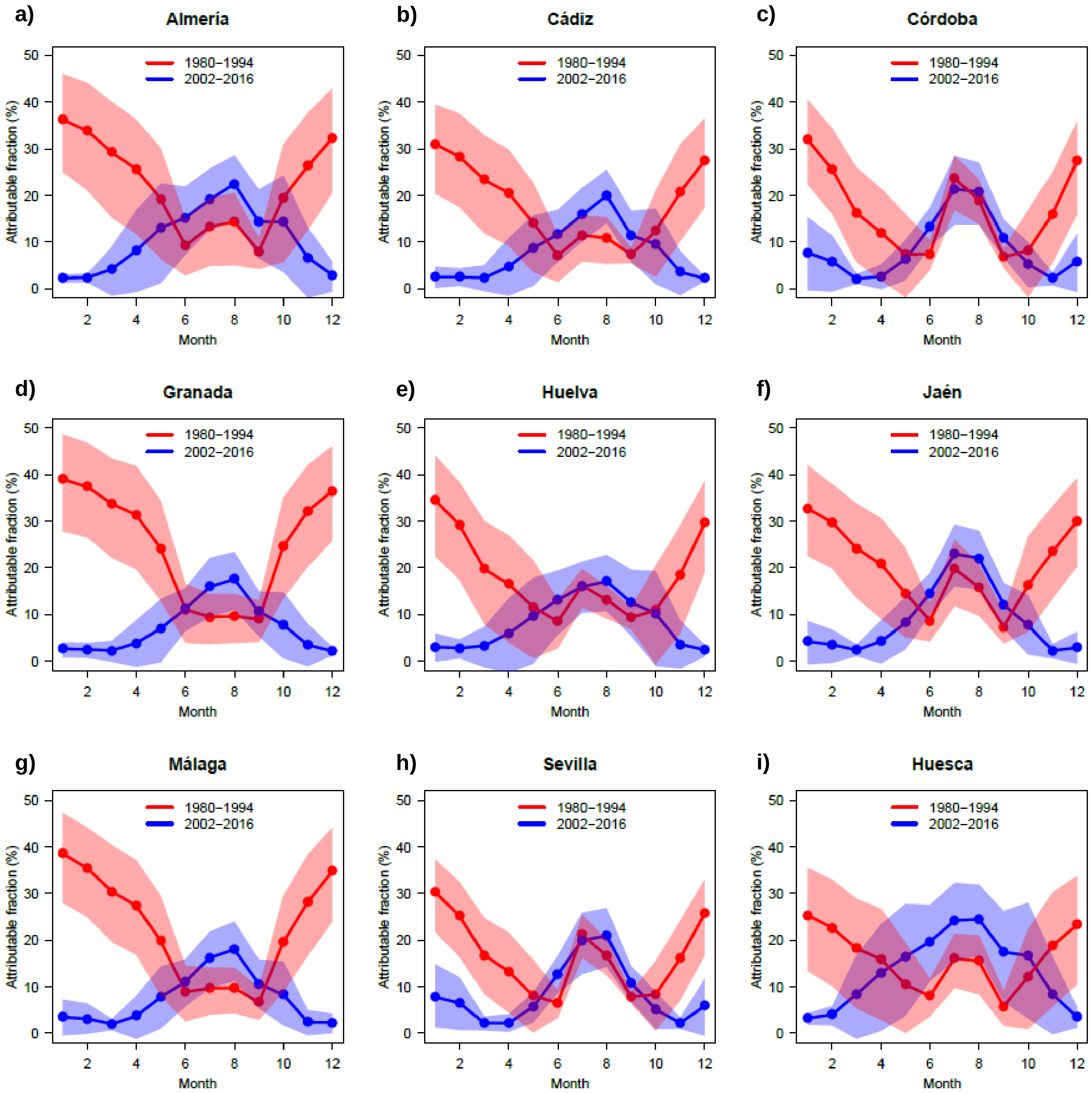

Supplementary Figure 33: **Monthly Attributable Fraction of respiratory disease mortality in the Spanish provinces.** The attributable fractions (AF) are estimated with the corresponding 95% empirical CI. The Minimum Mortality Temperature (MMT) of a given subperiod is used as the centring temperature of the subperiod ("*varying centring temperature assumption*", see Methods).

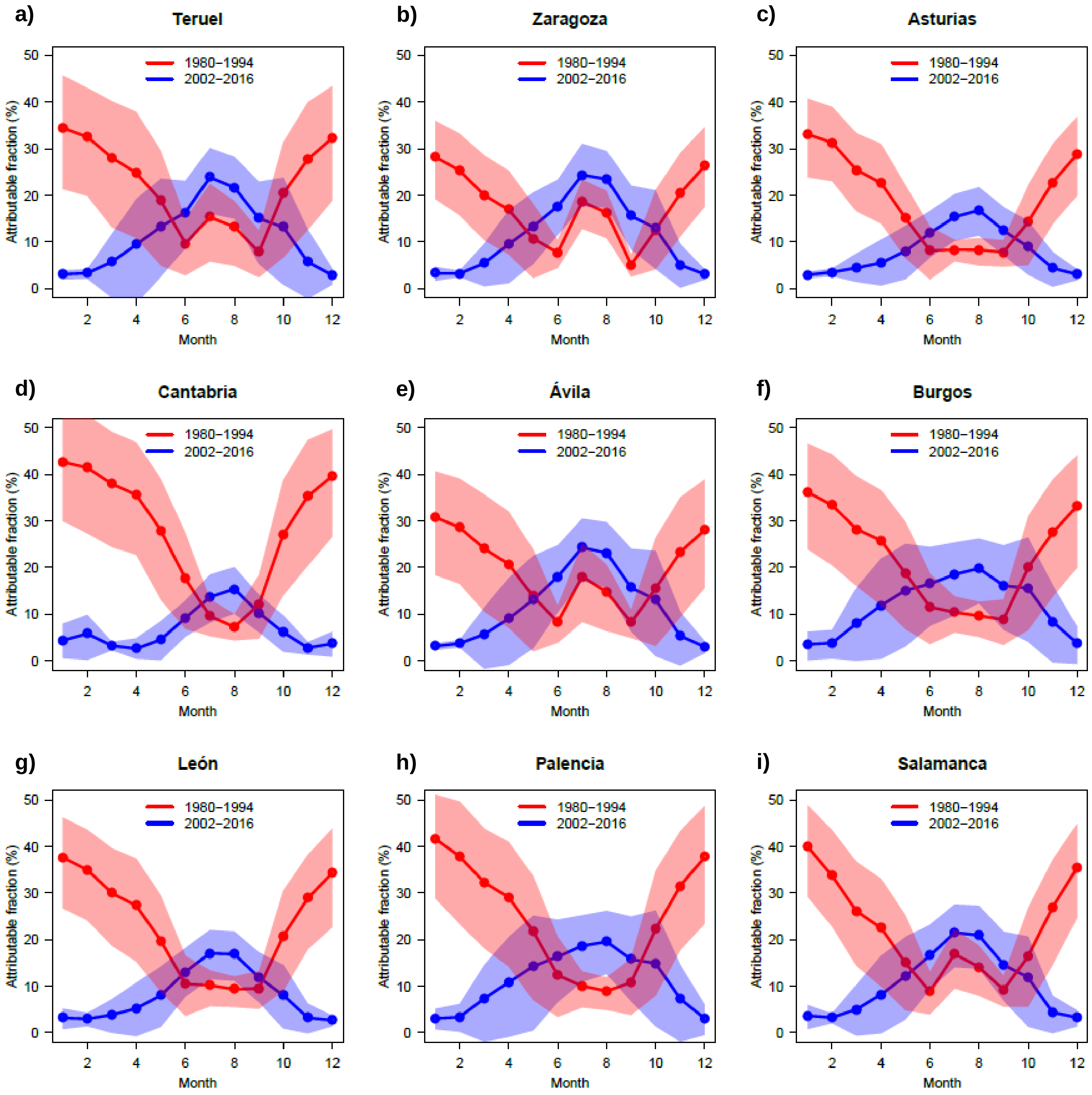

Supplementary Figure 34: **Monthly Attributable Fraction of respiratory disease mortality in the Spanish provinces.** The attributable fractions (AF) are estimated with the corresponding 95% empirical CI. The Minimum Mortality Temperature (MMT) of a given subperiod is used as the centring temperature of the subperiod ("*varying centring temperature assumption*", see Methods).

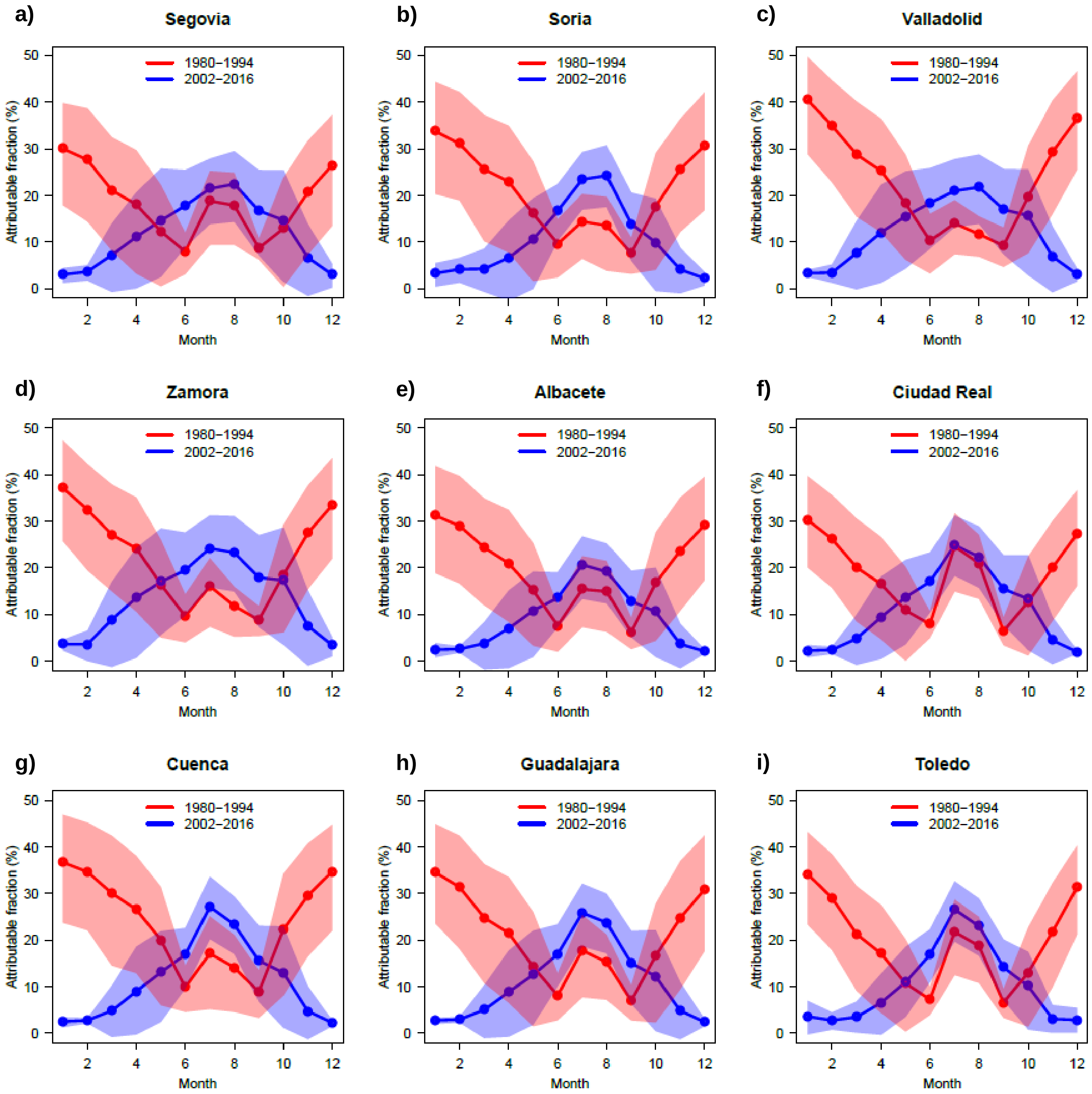

Supplementary Figure 35: **Monthly Attributable Fraction of respiratory disease mortality in the Spanish provinces.** The attributable fractions (AF) are estimated with the corresponding 95% empirical CI. The Minimum Mortality Temperature (MMT) of a given subperiod is used as the centring temperature of the subperiod (“*varying centring temperature assumption*”, see Methods).

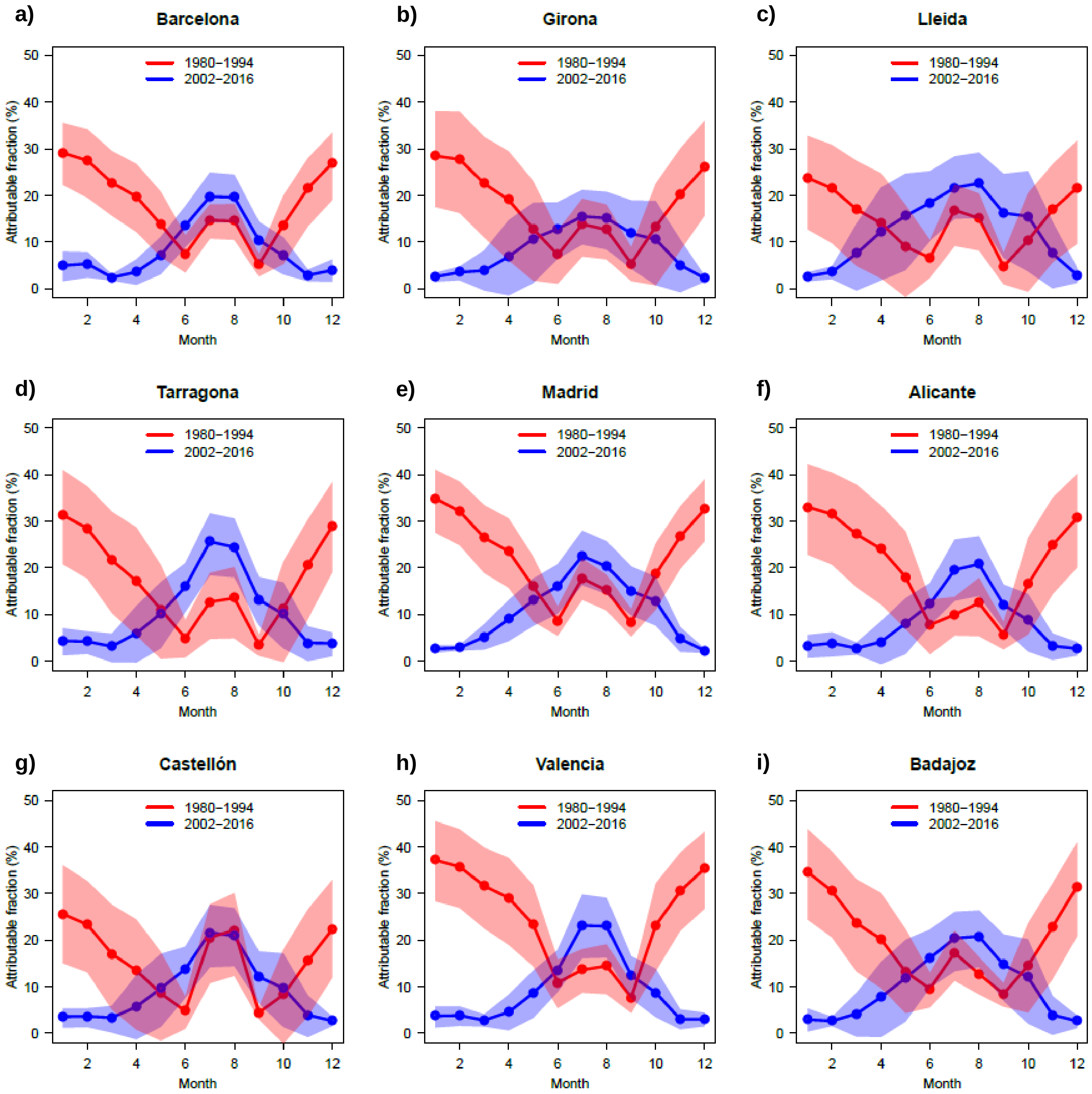

Supplementary Figure 36: **Monthly Attributable Fraction of respiratory disease mortality in the Spanish provinces.** The attributable fractions (AF) are estimated with the corresponding 95% empirical CI. The Minimum Mortality Temperature (MMT) of a given subperiod is used as the centring temperature of the subperiod (“*varying centring temperature assumption*”, see Methods).

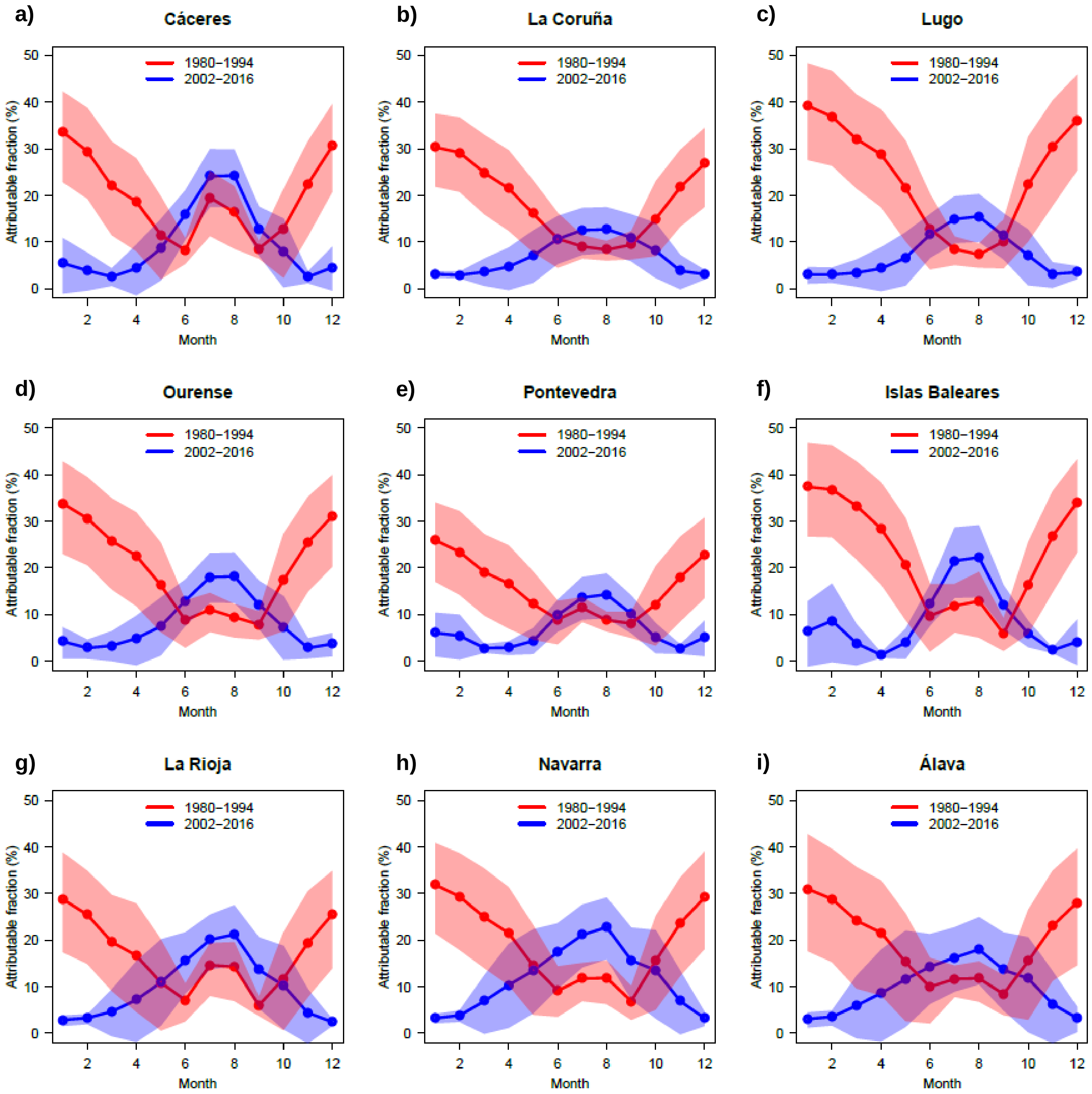

Supplementary Figure 37: **Monthly Attributable Fraction of respiratory disease mortality in the Spanish provinces.** The attributable fractions (AF) are estimated with the corresponding 95% empirical CI. The Minimum Mortality Temperature (MMT) of a given subperiod is used as the centring temperature of the subperiod (*"varying centring temperature assumption"*, see Methods).

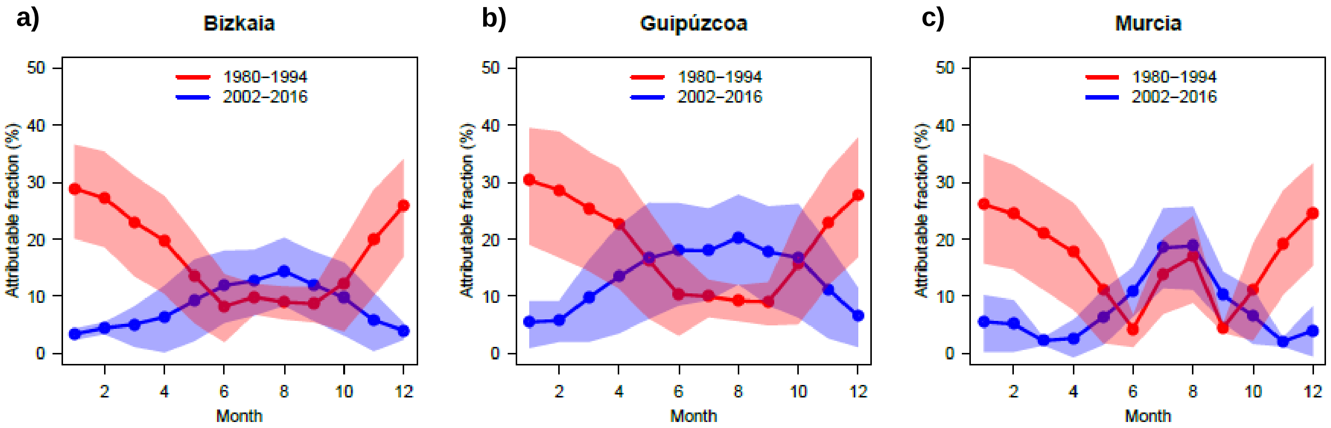

Supplementary Figure 38: **Monthly Attributable Fraction of respiratory disease mortality in the Spanish provinces.** The attributable fractions (AF) are estimated with the corresponding 95% empirical CI. The Minimum Mortality Temperature (MMT) of a given subperiod is used as the centring temperature of the subperiod ("*varying centring temperature assumption*", see Methods).

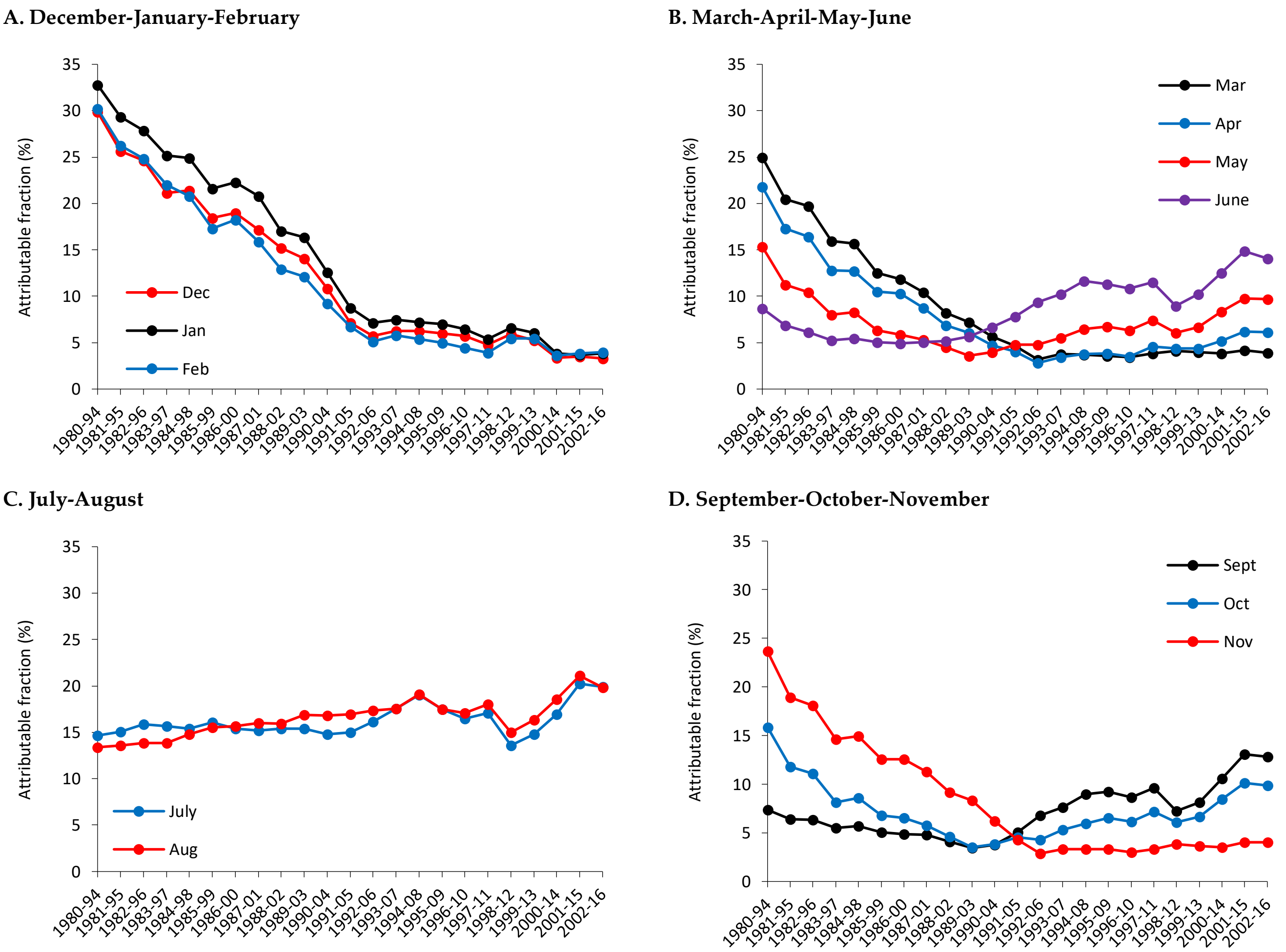

Supplementary Figure 39: **Evolution of the monthly Attributable Fraction of respiratory disease mortality in Spain.** The Minimum Mortality Temperature (MMT) of a given subperiod is used as the centring temperature of the subperiod (*"varying centring temperature assumption"*, see Methods).

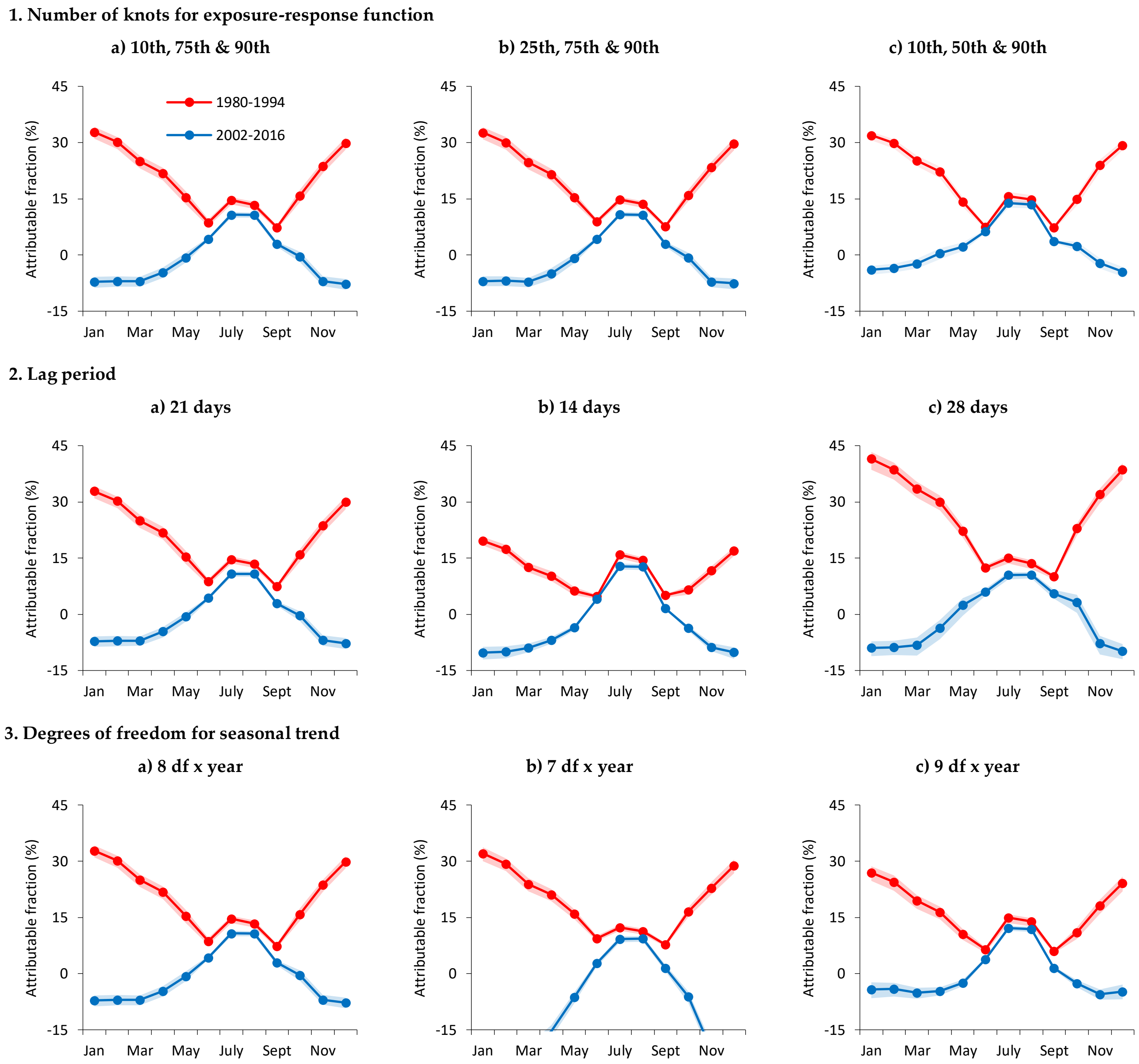

Supplementary Figure 40: **Sensitivity analysis on the monthly Attributable Fraction of respiratory disease mortality in Spain.** The attributable fractions (AF) are estimated with the corresponding 95% empirical CI.
